# Harnessing genetic engineering to drive economic bioproduct production in algae

**DOI:** 10.3389/fbioe.2024.1350722

**Published:** 2024-01-29

**Authors:** Abhishek Gupta, Kalisa Kang, Ruchi Pathania, Lisa Saxton, Barbara Saucedo, Ashleyn Malik, Yasin Torres-Tiji, Crisandra J. Diaz, João Vitor Dutra Molino, Stephen P. Mayfield

**Affiliations:** ^1^ Mayfield Laboratory, Department of Molecular Biology, School of Biological Sciences, University of California San Diego, San Diego, CA, United States; ^2^ California Center for Algae Biotechnology, University of California San Diego, San Diego, CA, United States

**Keywords:** sustainability, biotechnology, algae, genetic engineering, bioproducts, biofuels, transcription factors

## Abstract

Our reliance on agriculture for sustenance, healthcare, and resources has been essential since the dawn of civilization. However, traditional agricultural practices are no longer adequate to meet the demands of a burgeoning population amidst climate-driven agricultural challenges. Microalgae emerge as a beacon of hope, offering a sustainable and renewable source of food, animal feed, and energy. Their rapid growth rates, adaptability to non-arable land and non-potable water, and diverse bioproduct range, encompassing biofuels and nutraceuticals, position them as a cornerstone of future resource management. Furthermore, microalgae’s ability to capture carbon aligns with environmental conservation goals. While microalgae offers significant benefits, obstacles in cost-effective biomass production persist, which curtails broader application. This review examines microalgae compared to other host platforms, highlighting current innovative approaches aimed at overcoming existing barriers. These approaches include a range of techniques, from gene editing, synthetic promoters, and mutagenesis to selective breeding and metabolic engineering through transcription factors.

## 1 Introduction

Humans have cultivated plants as a sustainable source of food, medicine, and materials for millennia. Since the first Agricultural Revolution (10,000 BC), we have optimized our agricultural practices to meet the increasing demands of our civilization (Harlander, 2002). Today, with growing populations and food production shortcomings brought about by climate change, we can no longer count on the traditional crop optimization cycles to keep the world fed. According to the United Nations, the world population is expected to increase to about 10 billion by 2059 (World Population Prospects, 2022). Over-exploitation of arable land, rising global temperatures, changing climate, and extreme weather make land crops an increasingly strained source of food, feed, and energy (Kurukulasuriya and Rosenthal, 2013). Hence, new technology and resources are essential to meet the needs of future generations.

Microalgae hold significant promise as a sustainable and renewable source of food, feed, and energy (Barbosa et al., 2023). Microalgae are microscopic photosynthetic organisms that have high growth rates, can be cultivated using non-arable land and non-potable water, and have the ability to produce a variety of bioproducts, such as food supplements, biofuels, biopolymers, nutraceuticals, animal feeds, and medical therapeutics (Khan et al., 2018; Dolganyuk et al., 2020; Torres-Tiji et al., 2020; Diaz et al., 2023). Additionally, microalgae capture and utilize carbon dioxide (CO_2_) from the atmosphere to make these products, helping to mitigate greenhouse gas Emissions (Onyeaka et al., 2021). With their versatile bioproducts production capabilities, ability for carbon sequestration, and capacity to do this using non-arable land and non-potable water, microalgae offer a promising avenue for meeting society’s future demands, while reducing environmental impacts associated with this increased production. However, despite their immense potential, the lack of concerted domestication efforts has resulted in relatively expensive biomass production. This chicken or egg problem, where large-scale cultivation is needed to achieve low-cost production, and low-cost production is needed for large-scale utilization, has slowed the rate at which algae will attain widespread utilization (Chen and Wang, 2022). Overcoming the initial cost barriers will be crucial to fully exploit the advantages microalgae offer and establish them as low-cost, sustainable, and scalable solutions for the future (Lane, 2022).

Past research endeavors have demonstrated continuous improvements in a number of the properties of algae cultivation, aiming to either boost biomass production or optimize the downstream process (Chu, 2017; Maity, 2019; Kang et al., 2022; Chettri et al., 2023). Several methods have been utilized to enhance biomass production, including improved pond design, improved crop protection, better growth media, and water chemistry, improving photosynthetic efficiency, working with extremophile strains, and optimizing strain development through molecular engineering, breeding, selection, and *in-vitro* evolution. For enhanced metabolic engineering, multiple techniques are available, one of which involves either overexpressing or repressing functional genes (Mochdia and Tamaki, 2021; Chettri et al., 2023). Earlier literature has surveyed these landscape of engineering tools for algae (Mochdia and Tamaki, 2021; Sproles et al., 2021; Dhokane et al., 2023; Khoo et al., 2023; Patel et al., 2023). In our current review, we update and expand upon these evaluations, providing fresh insights into the field. Our discussion starts with a comparison of microalgae against alternative production platforms, emphasizing new methods intended to improve the quality of microalgae-derived biomass. We delve into various methodologies such as gene editing, the introduction of synthetic promoters, mutagenesis, selective breeding, adaptive laboratory evolution, and metabolic engineering driven by transcription factors. We also present a thorough survey of studies focused on transcription factor-mediated metabolic engineering in microalgae. Additionally, we confront the existing hurdles and forecast potential developments, stressing the crucial integration of these innovative tools into commercially valuable algae strains.

## 2 Microalgae for sustainable bioeconomy

### 2.1 Overview of bioeconomy and existing production platforms

Today, we utilize renewable biological resources for food, materials, and energy, which offers an alternative to fossil resource-based economies (Bugge et al., 2016). A sustainable bioeconomy is one that includes a stronger focus on sustainability, including energy utilization, reduced greenhouse gas emissions, more efficient water utilization, and overall a transformative change in resource production and consumption to a more environmentally focused alternative to the current fossil-based economy (Antar et al., 2021). The bioeconomy presently utilizes a diverse range of production organisms, encompassing bacteria, yeast, fungi, plants, and mammalian cells (Table 1; Antranikian and Streit, 2022; Hankamer et al., 2023; Jo et al., 2023; Kamaludin and Feisal, 2023; Navarrete and Martínez, 2020; Nguyen and Lee, 2021; Sarwar and Lee, 2023; Soong et al., 2023; Zhang et al., 2017).

**TABLE 1:** Comparative overview of host organisms in biotechnology: Advantages and Disadvantages.

Host organism	Advantages	Disadvantages
Bacteria	Rapid growth, high density, easy genetic modification, extensive research, cost-effective media, used in protein and antibiotic production	Lack of glycosylation, codon bias, endotoxin production in *E.coli* affecting therapeutics
Fungi	Nutrient recycling, symbiosis with plants, efficient in producing antibiotics and enzymes, fermentation in food and beverage industries	Limited genetic knowledge, underdeveloped genetic tools, health risks, food spoilage concerns
Mammalian Cells	Can produce complex proteins with specific post-translational modifications (PTMs), efficient in eukaryotic protein synthesis and processing	Maintenance and scalability issues, vulnerability to viral contamination, labor-intensive, expensive
Plants	Human pathogen-free, environmentally friendly, less waste, and energy consumption	Slow growth, competition with food production, complex protein extraction and purification, different PTMs patterns compare to mammalian cells
Microalgae	Photosynthetic, high biomass, diverse bioproducts, health benefits, uses non-arable land, wastewater nutrient recovery	Toxin production, complex cultivation and processing, need for more genetic tools

The table provides a comparative analysis of various host organisms–bacteria, fungi, mammalian cells, plants, and microalgae–used in biotechnology. It systematically outlines the advantages and disadvantages of each, offering insights into their respective efficacies and challenges in biotechnological applications.

Bacterial hosts are employed as cell factories in the production of fuel, recombinant proteins, vitamins, chemicals, and plasmid DNA (Gonçalves et al., 2012; Ferrer-Miralles and Villaverde, 2013; Acevedo-Rocha et al., 2019; Cho et al., 2022). Notably, *Escherichia coli* is a popular choice in biomanufacturing due to its rapid growth, well-characterized genetic makeup, and the ease with which it can be genetically manipulated. It is commonly used for producing proteins, antibiotics, and small molecules, due to its cost-effectiveness, versatility, and rapid growth on various nutrients (Blount, 2015). It is a key model organism in molecular biology, contributing to understanding genetic code, replication, transcription, and translation. However, it is less suitable for complex proteins requiring specific post-translational modifications (PTMs) (Corchero et al., 2013). *Escherichia coli* produces about 30% of therapeutic proteins but lacks PTMs like glycosylation and phosphorylation, crucial for many protein therapies (Baeshen et al., 2015). Efforts are ongoing to engineer *E. coli* strains to overcome this limitation, but currently, complex proteins are mainly produced using mammalian cell culture. *Escherichia coli* is also utilized in biofuel and bio-alcohol production, benefiting from its ability to grow in different conditions and its high growth and metabolism rates (Chen et al., 2013; Koppolu and Vasigala, 2016; Wang et al., 2017; Liang et al., 2020). Challenges include processing cheap raw materials like cellulosic and hemicellulosic hydrolysates, which can contain growth-inhibiting toxic compounds and cause osmotic stress when using concentrated sugars (Koppolu and Vasigala, 2016).

The fungal kingdom, encompassing yeasts to filamentous fungi, excels in producing bio-based products like enzymes, acids, and pharmaceuticals (Meyer et al., 2016; Sanchez and Demain, 2017; Corbu et al., 2023). Species like *Aspergillus*, *Trichoderma reesei*, and *Saccharomyces cerevisiae* are key in recombinant protein and industrial enzyme production (El-Gendi et al., 2021; Lübeck and Lübeck, 2022). Fungi also generate sustainable biomaterials, food ingredients with prebiotic benefits, and aid in fermentation in food and beverage industries (Patel et al., 2016; Singdevsachan et al., 2016; Wösten, 2019). Agriculturally, they enhance crop growth through nutrient uptake symbiosis (Wu et al., 2022). However, some fungi pose health threats (Fisher et al., 2020). Despite their potential, our knowledge of fungal genetics, metabolism, and physiology is limited, necessitating advanced tools for better utilization (Naranjo-Ortiz and Gabaldón, 2020; El Enshasy, 2022). Challenges in large-scale fungal cultivation and product recovery remain, requiring further research for improved productivity and cost-efficiency in fermentation.

Mammalian cell lines like Chinese hamster ovary (CHO) cells, baby hamster kidney (BHK21) cells, and murine myeloma cells (NS0 and Sp2/0) are preferred for biopharmaceuticals, particularly for complex proteins with specific PTMs (Dumont et al., 2016; Arnau et al., 2019). These cells secrete proteins directly, avoiding the need for cell lysis and protein refolding required in bacterial production. However, non-human mammalian cells might introduce non-human PTMs, potentially causing antibody responses in humans (Ghaderi et al., 2010). While crucial for therapeutic proteins and vaccines, mammalian cell cultures face challenges like maintenance, scalability, susceptibility to viral contamination, and decreased viability with successive passages due to genetic changes (Li et al., 2010; Seth, 2012; Barone et al., 2020). Mammalian cell-based production, though effective, can be labor-intensive and expensive.

Plants are crucial for a sustainable bioeconomy, being the sole sustainable source for food, feed, fiber, renewable fuels, pharmaceuticals, and carbon sequestration (Vanholme et al., 2013; Munir et al., 2022). They support all life as primary producers and are key for fermentation due to their starch and sugar content. However, utilizing plants for the production of biofuels may pose risks to forest areas and biodiversity, potentially escalate food prices, and create a strain on water resources (Koh and Ghazoul, 2008; Furtado et al., 2014; Ramos et al., 2016). Moreover, plants face challenges from environmental stressors like climate change, reducing yields, and causing economic losses (Dhankher and Foyer, 2018; Zaidi et al., 2020). Genetic engineering and traditional breeding improve crop resilience and nutritional content but raise concerns like cross-pollination and resistance to pests (Van Acker et al., 2007; Bawa and Anilakumar, 2013). Intense cultivation also leads to environmental issues like eutrophication and biodiversity loss (Tilman, 1999; Schütte et al., 2017; Li et al., 2020). Balancing the benefits and impacts of plant-based bioeconomy is a significant ongoing challenge.

### 2.2 Microalgae as a platform for sustainable bioeconomy: Potentials and current challenges

Microalgae have emerged as a promising platform for the sustainable bioeconomy, due to their unique characteristics and versatile applications, and the fact that they do not compete with traditional crop cultivation (Khan et al., 2018). Compared to bacteria, fungi, and mammalian cells, microalgae offer several advantages in the context of sustainable bioproduct production (Table 1). Microalgae exhibit photosynthetic capabilities, enabling them to transform light and CO_2_ into organic carbon products, including proteins, lipids, and carbohydrates (Rasala and Mayfield, 2015). Microalgae possess a remarkable capacity to generate a diverse range of bioproducts, encompassing food supplements, biofuels, biopolymers, nutraceuticals, animal feeds, and medical therapeutics (Khan et al., 2018; Nur and Buma, 2019; Dolganyuk et al., 2020; Torres-Tiji et al., 2020; Diaz et al., 2023). Additionally, in comparison to plants, their biomass production offers several advantages, including rapid growth, lack of competition for resources used by crops, higher yields, metabolic diversity, utilization of non-arable land, nutrient recovery from wastewater, efficient carbon capture, and accelerated development of new production strains (Rasala and Mayfield, 2015; Fu et al., 2016; Benedetti et al., 2018).

However, achieving economic viability for microalgae-based bioproducts remains a challenge. The optimization of cultivation, harvesting, extraction, and downstream processing costs will all be required to ensure the competitiveness of these products against traditional sources. Although microalgae offer great genetic diversity, there is still a significant deficit in the number of sequenced genomes and the number of microalgae that have been genetically transformed in the lab (Lin et al., 2019; Maréchal, 2021). The full potential of microalgae is still largely unrealized due to our limited understanding of their metabolic pathways, regulatory networks, and genetic makeup (Kumar et al., 2020). At the commercial scale, growing microalgae in open ponds is a challenging task due to the high risk of biological contamination (Lam et al., 2018). Additionally, the high cost of downstream processing must be reduced to make microalgae a platform capable of producing products with commodity pricing (Khoo et al., 2020). All of these bottlenecks can be surmounted, albeit with substantial investments of time and resources. However, such investments are imperative for preserving our current standard of living without exacerbating the degradation of the remaining environment on this planet.

### 2.3 Current strategies to engineer microalgae

**FIGURE 1:**
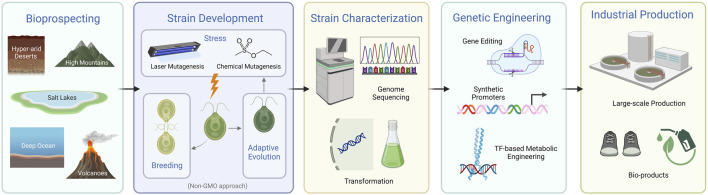
Overview of strategies for establishing microalgae as an industrial platform (created with BioRender.com). The schematic provides a streamlined depiction of the key phases in developing microalgae for industrial applications, starting from bioprospecting and culminating in large-scale industrial production. It highlights the progression through strain development, characterization, and genetic engineering.

Current endeavors to engineer microalgae primarily aim to bolster their economic viability for bioproducts and biofuel production (De Bhowmick et al., 2015; Chu, 2017; Lin et al., 2019; Kumar et al., 2020; Mochdia and Tamaki, 2021; Sproles et al., 2021; Chettri et al., 2023). This journey begins with bioprospecting, the systematic search for novel and robust microalgal strains (Barclay and Apt, 2013). The discovery and analysis of new species invariably lead to the unveiling of new genomes and genes, enabling the identification of phenotypes that better align with biotechnological needs. Once a new strain is identified, attention pivots towards enhancing phenotypes via mutagenesis, breeding, adaptive laboratory evolution, stress resistance, and then through genetic engineering and gene editing techniques, all capable of tailoring a microalgae’s genetic makeup to fulfill desired phenotypic objectives (Fields et al., 2019; Kumar et al., 2020; LaPanse et al., 2021). Genome sequencing is commonly employed to provide a foundational understanding of the genetic blueprint of microalgae, facilitating future targeted genetic modifications. Genetic manipulation techniques have been advanced to fine-tune the genome of many microalgae. This allows for the introduction or enhancement of specific traits that can significantly boost yield and cut production expenses. Many studies have underscored the use of genetic engineering in different microalgae species to enhance the production of bioproducts such as lipids, pharmaceutical proteins, and carotenoids, among others (Larkum et al., 2012; Patel et al., 2019; Grama et al., 2022). In parallel, researchers have crafted synthetic promoters to fine-tune gene expression, ensuring optimal production of targeted compounds (Milito et al., 2023). Additionally, significant efforts have been made to boost the overall photosynthetic efficiency of microalgae, aiming to increase biomass productivity (Kumar et al., 2021). These collective strategies, from the discovery of novel strains to genetic manipulation, serve as important building blocks in advancing microalgae as a more economical and sustainable solution within the burgeoning bio-economy (Figure 1). In the subsequent subsections, a more detailed analysis of each of these strategies will be provided, examining the comprehensive approaches employed to optimize the productivity and resilience of microalgae for industrial applications.

#### 2.3.1 Bioprospecting

Accelerating microalgal technology development involves using naturally resilient algae strains. Bioprospecting, exploring the diversity of over 50,000 microalgae species, identifies strains with beneficial traits like high lipid content and environmental robustness (Guiry, 2012; Barclay and Apt, 2013; Morales et al., 2021). This approach leverages natural variation to find strains meeting biotechnological goals, avoiding the complexities and costs of genetic engineering. Bioprospecting efficiently finds suitable strains, simplifying the process compared to engineering desired traits into less suitable species.

Several bioprospecting projects have been conducted to identify and isolate highly productive strains (Araujo et al., 2011; Bohutskyi et al., 2015; Neofotis et al., 2016; Silveira Júnior et al., 2019; Archer et al., 2021; Grubišić et al., 2022; Maity and Mallick, 2022; Saeed et al., 2022; Stirk and van Staden, 2022). For instance, Neofotis et al. (2016), discuss a bioprospecting project aimed at identifying microalgal strains suitable for biofuel production, focusing on high growth rates and high lipid content. Promising strains include coccoid green algae like *Acutodesmus obliquus* and *Chlorella sorokiniana*, as well as *Desmodesmus*, *Ankistrodesmus*, and *Coelastrella* strains. These findings enrich the biological resources for algae-based biofuel production and have been tested successfully in outdoor ponds (Neofotis et al., 2016). However, a key limitation of bioprospecting is the need for effective methods to identify desired traits in numerous microalgal species, a challenging and resource-intensive task like finding a needle in a haystack (Stirk and van Staden, 2022). Efficient screening is essential to optimize bioprospecting’s effectiveness and cost-efficiency in the advancement of microalgal technology.

#### 2.3.2 Mutagenesis, breeding, adaptive laboratory evolution, and stress resistance

After bioprospecting isolates algae with specific traits, mutagenesis and breeding can enhance these traits. Mutagenesis alters genetic information through natural or artificial means, causing DNA mutations, while breeding involves controlled mating for desired characteristics (Larkum et al., 2012; Torres-Tiji et al., 2020; Trovão et al., 2022; Diaz et al., 2023). These non-genetically modified organisms (non-GMO) methods, used in agriculture for centuries, enhance microalgal traits without adding foreign DNA, avoiding GMO regulations (Benedetti et al., 2018). The application of random mutagenesis has proved to be a robust and effective tool for generating desirable traits within microalgae strains, and microalgae can be rapidly cultured, mated, and selected for strains exhibiting higher bioproduct yields within just a few weeks time (Trovão et al., 2022; Diaz et al., 2023). Various case studies underscore the potential of mutagenesis in enhancing traits essential for industrial applications. For instance, using laser mutagenesis, the biomass of two third-generation Chlorella strains, FACHB 9 and FACHB 31, significantly increased, showcasing a potential avenue for biomass enhancement via physical mutagenesis (Xing W et al., 2021). Similarly, in a chemical mutagenesis example, Nayak et al. (2022) utilized ethyl methanesulfonate (EMS) mutagenesis coupled with fluorescence-activated cell sorting (FACS) based screening to generate a *Chlorella* sp. *HS2* mutant with higher lipid content and productivity compared to the non-mutagenized and wild strains (Nayak et al., 2022). In a separate research conducted by Fields et al. (2019) a tactical combination of mutagenesis and genome shuffling was utilized to significantly amplify the expression of green fluorescent protein (GFP) by 15-fold, all without altering the GFP gene or compromising the growth rate of the strain (Fields et al., 2019). Numerous other successful instances of utilizing mutagenesis to amplify desired traits, such as thermotolerance, carotenoid content, and the creation of starchless mutants, have been documented (Trovão et al., 2022; Diaz et al., 2023). Breeding and mutagenesis, enhanced by high-throughput screening, are key in microalgae trait improvement. Their gene-agnostic nature allows broad genetic changes, potentially yielding new beneficial traits. However, the randomness of mutations requires luck, yet high-throughput screening increases the odds of finding useful mutations. Effective screening is essential to navigate genetic diversity and maximize these methods’ potential in microalgae industrial applications.

Building on the potential of mutagenesis and breeding for enhancing microalgal traits, adaptive laboratory evolution (ALE) offers another avenue for genetic modification and trait improvement under specific environmental pressures (Arora et al., 2020; Sun et al., 2018a). This generally involves growing a strain under conditions containing specific selective pressures over time. This allows for the development of new traits as the strain adapts and evolves to the provided selective pressure. Previous research has provided a framework for these experimental setups while highlighting the importance of consistent stress placed on cultures and the advantages of automated cultivation (LaPanse et al., 2021). ALE methods have been used to effectively enhance *C. reinhardtii* in various aspects of growth and efficiency. Successful experiments have been conducted for the enhancement of metabolic pathways, bioproducts, increased uptake of specific substrates, tolerance to abiotic stress, and biofuel production to name a few (LaPanse et al., 2021; Zhang et al., 2021). This has led to the increase of desirable natural products such as terpenoids, carotenoids, and lipids (Fu et al., 2013; Arora et al., 2020; Jia et al., 2023). ALE can also be used in the reverse as a method to improve the degradation of particular compounds such as phenols, while still increasing biomass (Wang et al., 2016). Another aspect of these processes are stress resistance/tolerance studies that take a strain that is evolved or already capable of producing a desired product and placing it under stress conditions that increase the yield of that product or process (Chen et al., 2017). This process can be considered “stress modulation by process engineering” whereby stress conditions are optimized for the production of a particular product. The stress conditions are generally implemented as environmental stressors such as light exposure and temperature, oxidative stress, or modified nutrients such as nitrogen starvation, low salinity, and modified glucose levels (Chen et al., 2017; Chu, 2017; Sun et al., 2018b). While the success of these experiments highlights the inherent adaptability of microalgae, they underscore the future need for transcriptional engineering to advance the yield of products or pathways that may not occur fast enough through ALE and stress selection.

#### 2.3.3 Genome sequencing and transformation method

The journey of uncovering and examining new species through bioprospecting, along with trait augmentation via mutagenesis and breeding, invariably results in the disclosure of new genomes with unique genetic architectures. This prompts the need to unravel the genetic blueprint of microalgae. Over the past decade, there has been a marked rise in the number of genomes that have been sequenced. Moreover, numerous sequencing projects are currently underway with the aim of sequencing hundreds more sequences. Additionally, a number of transcriptomics, proteomics, and metabolic studies have been conducted to gain insights into strain improvement strategies (Chettri et al., 2023). Kumar et al. (2020) have assembled a roster of microalgae species with sequenced genomes and current ongoing sequencing projects (Kumar et al., 2020).

Numerous methods have been established for effectively modifying the genetic composition of microalgae. These techniques encompass a range of approaches, including glass bead agitation, biolistic particle bombardment, Agrobacterium-mediated transformation, electroporation, silicon carbide whiskers, and nanoparticle-based methods (Jinkerson and Jonikas, 2015; Sproles et al., 2021; Chettri et al., 2023). For a comprehensive analysis of some of these methods, Ng et al. (2017) have provided a detailed comparison, taking into consideration factors such as cell wall removal and ease of use in their study (Ng et al., 2017). Additionally, Kumar et al. (2020) have extensively documented transformation techniques employed across various microalgae species (Kumar et al., 2020).

#### 2.3.4 Gene editing

Genomic advancements have enabled CRISPR-Cas9 for precise genetic editing in microalgae, outperforming traditional methods (Ng et al., 2017; Aratboni et al., 2019; Naduthodi et al., 2019; Ghribi et al., 2020; Kelterborn et al., 2022). This technology allows specific gene modifications, advancing microalgal biotechnology (Shin et al., 2016; Greiner et al., 2017; Patel et al., 2019; Lee et al., 2023). The approach is notably successful in microalgae species like *C. reinhardtii*, addressing gene expression challenges, including the toxicity from continuous Cas9 protein presence and unintended mutations stemming from Cas9 expression via vectors, by employing preassembled Cas9-ribonucleoproteins (Cas9-RNPs) that are delivered through electroporation (Jiang et al., 2014; Aratboni et al., 2019; Kelterborn et al., 2022; Dhokane et al., 2023; Jeong et al., 2023). Preassembled Cas9-RNPs eliminate the need for Cas9 expression by the host cell, and reduce the risk of generating off-target mutants, and re-editing a previously modified target site (Kelterborn et al., 2022). Patel et al. (2019) provided an overview of several studies that utilized CRISPR-Cas9 for genome editing in green algae and diatoms (Patel et al., 2019).

A notable example of gene editing with CRISPR is the delivery of Cas-RNPs and an editing template into *Nannochloropsis oceania* IMET1 via electroporation (Naduthodi et al., 2019). The authors achieved highly efficient homology-directed repair (HDR). The Cas9/single guide RNA (sgRNA) RNP delivery enhanced the HDR at the nitrate reductase (NR) target site, generating ∼70% of positive mutant lines, indicating a significant improvement in editing efficiency compared to the native HDR system alone. Additionally, Lin et al. (2021) developed a novel approach to designing sgRNA via 20 guanines, called Adaptive Single Guide Assisted Regulation DNA (ASGARD) and coupled it with the dCas9 system in *C. sorokiniana*. Among the transformants, this approach led to an increase in protein content, reaching up to 60% (w/w) of DCW, with the highest protein concentration being 570 mg/L (Lin et al., 2021). In another study, the authors developed a CRISPR-Cas9 reverse-genetics pipeline and used it to identify a TF that regulates lipid accumulation, called ZnCys, in *Nannochloropsis gaditana* (CCMP 1894) (Ajjawi et al., 2017). By modulating the expression of ZnCys via Cas9-mediated insertional attenuation in the 5’ UTR and RNAi, Ajjawi et al. (2017) discovered that the lipid productivity of *ZnCys*-RNAi-7 doubled in the absence of changes in the components of the triacylglycerol (TAG) synthesis pathway. In a different study conducted by Baek et al. (2016a) the utilization of CRISPR-Cas9 led to the knockout of two genes, resulting in a strain that constitutively produces zeaxanthin and demonstrates enhanced photosynthetic productivity (Baek et al., 2016a). CRISPR enhances gene expression in microalgae like *C. reinhardtii* by targeting specific genome sites, contrasting the randomness of traditional DNA insertion methods (Zhang R et al., 2014; Kim et al., 2020). It improves recombinant gene expression and can knock out genes impeding this process. This precise genome editing creates strains with advanced recombinant expression, advancing microalgal biotechnology for industrial uses.

#### 2.3.5 Synthetic promoters

Efforts to enhance microalgae bioproduct production have led to the development of native, hybrid, and synthetic promoters (Kumar et al., 2020). Despite efficient DNA silencing and limited native promoters, researchers have made progress with endogenous promoters in the model green alga *C. reinhardtii*. These come from genes like the Rubisco small subunit (RBCS2), heat shock protein 70A (HSP70A), photosystem I subunit D (PSAD), glutamate dehydrogenase gene (GDH2), and the acyl carrier protein gene (ACP2). They have also created synthetic ones like the HSP70A-RBCS2 fusion (AR) and the GDH2-ACP2 fusion (GA) (Cerutti et al., 1997; Schroda et al., 2002; Scranton et al., 2016; Schroda, 2019; Neupert et al., 2020; Chen et al., 2023; Milito et al., 2023). GA showed seven times higher expression than AR, responding well to blue light. However, exogenous protein accumulation remains low in microalgae, with the highest levels in chloroplast-expressed genes reaching up to 5% of total soluble protein in *C. reinhardtii*, below the economic threshold for recombinant products (Manuell et al., 2007; Scranton et al., 2016; Reyes-Barrera et al., 2021).

To tackle this lack of strong promoters, researchers have turned to synthetic biology. In a study by Baek et al., the light-inducible protein gene (LIP) promoter from *Dunaliella* was dissected to identify light-responsive elements for synthetic promoter construction in *C. reinhardtii* (Baek et al., 2016b). Two key motifs, the GT-1 binding motif and sequences over-represented in light-repressed promoters (SORLIPs), were identified within the 200 bp upstream region of the LIP gene. While the GT-1 motif alone did not significantly induce light-responsive gene expression, the SORLIP motifs, particularly when duplicated, significantly enhanced luciferase activity under medium and high light conditions. This led to the creation of a synthetic promoter with duplicated SORLIPs, demonstrating a stronger light-inducible response than the native LIP promoter, thereby offering a potent tool for controlled gene expression in microalgae under varying light intensities.

In another study, Scranton et al. (2016) created 25 synthetic promoters by analyzing RNA-seq data of the most highly expressed genes in *C. reinhardtii*, identifying key cis-regulatory elements (CREs), and strategically combining these elements with a core promoter sequence (Scranton et al., 2016). Among these, seven promoters drove the expression of the fluorescent protein mCherry over twice as high as the AR promoter. Their strongest, SAP-11, revealed the presence of the CCCAT motif within its sequence. This motif was identified as a core element in *C. reinhardtii*, being highly conserved and essential for promoter function in highly transcribed genes. The study also highlighted the significance of other motifs, such as AT-rich regions and TC-rich motifs, in the high-expressing gene promoters, indicating a unique promoter structure that contrasts with higher plant species. Building on this work, Einhaus et al. (2021) developed the AβSAP(i) promoter (Einhaus et al., 2021). This synthetic promoter results from the fusion of HSP70A and βTUB2 promoters, enriched with the RBCS2 first intron, which enhances gene expression in *C. reinhardtii*. It also incorporates strategically placed CREs identified by Scranton et al., including the CCCATGCA-motif (CCCAT) near −65 bp and the ATANTT-motif near the transcription start site (TSS). Modifications to these motifs, particularly the addition of the ATANTT motif at −130 and +15 bp positions, resulted in a significant increase in (E)-ɑ-bisabolene terpene production, achieving up to 2.5 mg/L culture volume and 3.2 mg/g cell dry weight (CDW).

Another effort by McQuillan et al. (2022), several novel CREs in native genes of *C. reinhardtii* were identified, leading to the development of a series of synthetic promoters named pCREs (McQuillan et al., 2022). Among them, the synthetic promoters pCRE-12 and pCRE-13 either matched or exceeded the expression levels of the AR promoter in the top 10% of transformants. This highlighted the variability of transgene expression, likely influenced by positioning effects, and underscored the importance of understanding and harnessing native regulatory elements in microalgae.

Given the notable advancements in synthetic promoter design over the past 2 decades, future efforts may be geared towards crafting highly potent synthetic promoters capable of fueling robust gene expression, a pivotal factor for boosting the output of valuable bioproducts. The employment of computational tools to pinpoint and fine-tune CREs amalgamated with synthetic core promoter sequences could lay the foundation for the emergence of a new class of potent and controllable synthetic algal promoters.

#### 2.3.6 Improving photosynthetic efficiency

A different approach to increasing microalgal biomass involves enhancing the efficiency of photosynthesis. While sunlight is the preferred option for large-scale outdoor raceway cultivation due to its abundance and emittance of a complete spectrum of light, its penetration is constrained below the surface, demanding extensive surface areas with shallow depths for sufficient biomass productivity (Ramanna et al., 2017). However, microalgal light utilization efficiencies and biomass productivity are reported to be higher under artificial light compared to sunlight. Despite the elevated operational costs associated with photobioreactors using artificial light, there are significant increases in biomass and intracellular metabolite productivity (Ramanna et al., 2017). To further enhance photosynthetic efficiency and overall biomass productivity in microalgal cultures, strategies such as mutagenesis, genetic engineering, and DNA insertional mutagenesis are employed (Vecchi et al., 2020; Kumar et al., 2021; Hu et al., 2023). Photosynthetic efficiency in microalgae is often hindered by photoinhibition, a response to excessive light exposure. Algae with truncated antenna systems are beneficial in mitigating this issue, as they absorb less light per cell, reducing the problem of supersaturation in the photosynthetic efficiency (Kumar et al., 2020; Kumar et al., 2021; Vecchi et al., 2020; Hu et al., 2023). Additionally, the process of non-photochemical quenching (NPQ) is a photoprotective mechanism that can be manipulated to increase biomass productivity in microalgae. Excess-absorbed light in NPQ is converted to heat, resulting in energy wastage. Vani et al. (2023) generated mutants of *C. reinhardtii* with shortened light-harvesting antennae that exhibited a lower NPQ, higher biomass, and higher PSII efficacy compared to the wild-type (Vani et al., 2023).

## 3 Transcription factors-based metabolic engineering

To optimize the production of desired compounds in microbial cell factories, traditional strategies focus on directly increasing, decreasing, or removing specific genes in metabolic pathways (Liu et al., 2013; Deng et al., 2022). However, due to the complexity of these pathways, such engineering may not always yield the expected results, with gene overexpression potentially causing an accumulation of harmful intermediates and gene suppression or removal potentially affecting cell growth. Transcription factors (TFs), which are proteins that regulate gene expression by binding to DNA, offer a powerful alternative by fine-tuning complex pathways at the transcriptional level. This method addresses the shortcomings of targeting individual genes and reduces the risk of fatal overexpression of multiple genes. Moreover, certain transcription factors can exert a broad influence, regulating several genes within a pathway to enhance the synthesis of target metabolites and bolster environmental resilience (Deng et al., 2022). For instance, in bacteria, metabolic engineering involving a handful of transcription factors has been demonstrated to effectively increase metabolite production (Cai et al., 2019; Tolibia et al., 2023).

TFs are also known to play a crucial role in shaping agronomic traits in crops. Research, particularly involving maize, has shown that certain TF-associated genes have undergone significant changes during domestication from its ancestral form, teosinte. These TFs influence key traits like seed size, resource partitioning, and corn ear development (Liu et al., 2020). Advances in next-generation sequencing (NGS) have enabled detailed analysis of maize genomes and their wild relatives, identifying genes and loci central to domestication. This research reveals TFs as drivers of notable phenotypic transformations, such as the evolution of maize kernels from enclosed in a hardened fruitcase to being exposed for consumption (Liu et al., 2020). Furthermore, in a study on tomatoes, introducing two snapdragon TFs significantly increased anthocyanin accumulation, enhancing antioxidant properties and imparting a deep purple hue to both the peel and flesh, similar to blackberries and blueberries (Butelli et al., 2008).

**FIGURE 2:**
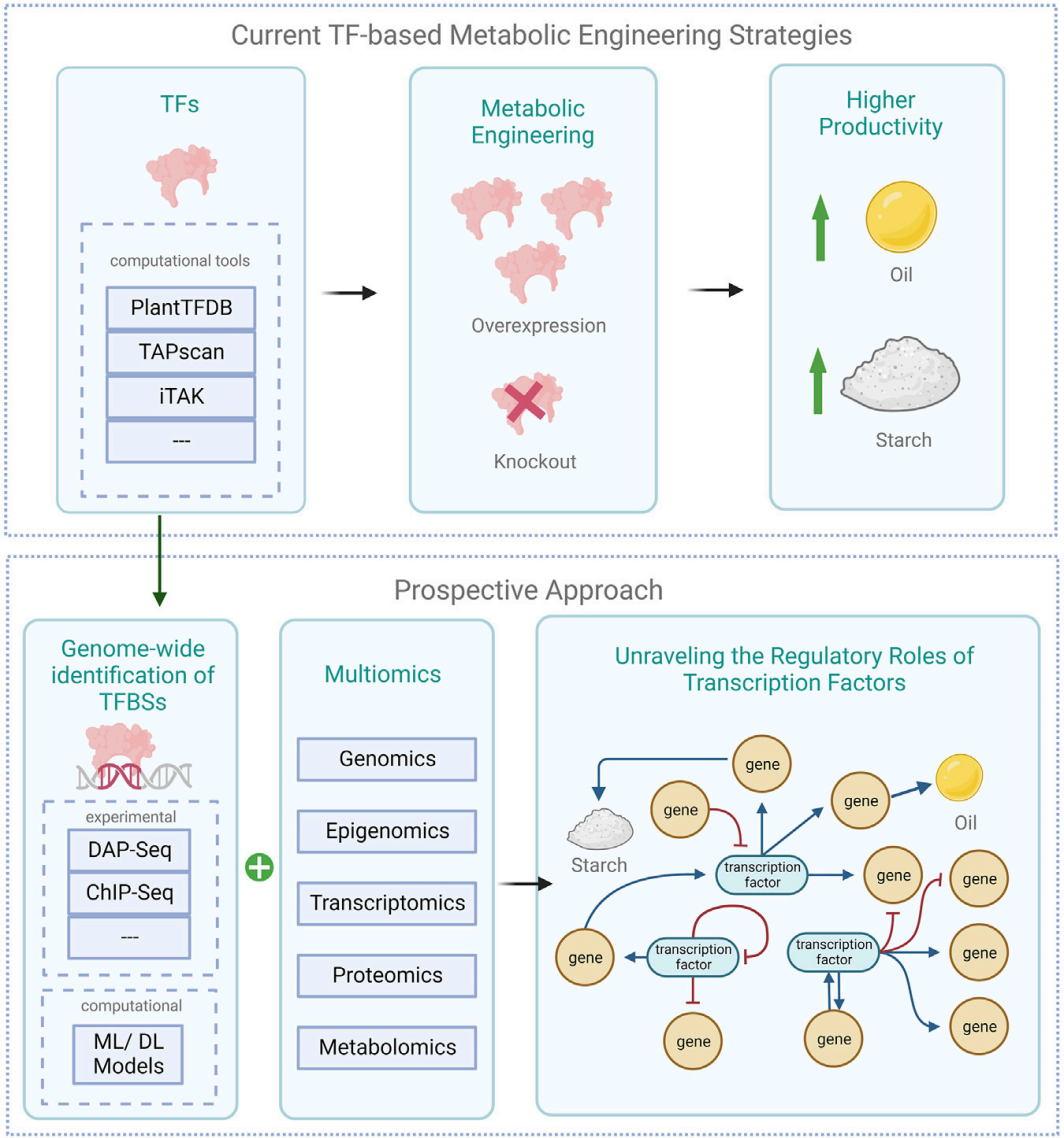
Schematic of current transcription factor-based engineering strategies in microalgae alongside prospective approaches (created with BioRender.com). The illustration presents current metabolic engineering strategies in microalgae using transcription factors, alongside prospective approaches. It highlights the genome-wide TF-TFBS pair identification via experimental and computational techniques, integrated with multiomics data, to decipher TFs’ regulatory functions in microalgae. DAP-seq denotes DNA Affinity Purification and sequencing; ChIP-Seq denotes Chromatin Immunoprecipitation sequencing; ML denotes machine learning, and DL deep learning models.

The use of transcription factor manipulation to engineer microalgae for sustainable bioproducts is a rapidly growing field of research. The identification, characterization, and genetic modification of transcription factors would enable the exploration of the vast potentials microalgae have to offer by improving the production of bioproducts, such as biofuels, pharmaceuticals, and carotenoids, while also increasing the efficiency of microalgae cultivation and improving the stress tolerance of microalgae (Sun et al., 2018b; Kwon et al., 2018; Bharadwaj et al., 2020; Choi et al., 2022). The following sub-sections provide an in-depth review of transcription factors and their binding sites within microalgae. This also includes a spotlight on studies that have utilized transcription-factor-based metabolic engineering to enhance the biosynthesis of lipids and carbohydrates (Figure 2).

### 3.1 Transcription factors in microalgae

The increasing availability of sequenced genomes in the past 2 decades has enabled the *in silico* identification of putative TFs, some of which are responsible for cellular processes, including cellular metabolism and responses to the environment (Mochdia and Tamaki, 2021). Computational methods and genome-wide comparative studies have been used to identify TFs across entire genomes in microalgae. Not only does this provide insight into the transcription factor families present, but it also facilitates the exploration of the evolutionary history of photosynthetic organisms (Thiriet-Rupert et al., 2016).

A number of studies have used computational pipelines to conduct a genome-wide identification of the TF complement in haptophytes (*Tisochrysis lutea*, *Emiliania huxleyi*, and *Pavlova* sp.), stramenopiles (the *Eustigmatophycea*, *Nannochloropsis gaditana*, and diatoms *Phaeodactylum tricornutum* and *Thalassiosira pseudonana*), green alga *C. reinhardtii*, and the red alga *Porphyridium purpureum* (Riaño-Pachón et al., 2008; Rayko et al., 2010; Thiriet-Rupert et al., 2016). Furthermore, an online resource named PlantTFDB provides information on transcription factors across 16 Chlorophyta species (Guo et al., 2008; Jin et al., 2017). Lang et al. (2010) introduce the TAPScan database, which encompasses extensive classification rules for transcription-associated proteins (TAPs; TFs, and Transcription Regulators) and has been utilized in genome-wide analyses of plants and algae (Lang et al., 2010). This database also outlines the Viridiplantae TAP dynamics timeline through phylogenetic comparative methods, while iTAK serves as another online tool for predicting and classifying TFs based on consensus rules from the literature (Zheng et al., 2016). PhycoCosm stands as another all-encompassing asset for algal research, serving as a repository of genomic data and offering comparative gene family profiles, including those encompassing transcription factor families (Grigoriev et al., 2021).

### 3.2 Transcription factors binding sites

Identifying transcription factor binding sites (TFBSs) in microalgae will be crucial for understanding the regulatory mechanisms that govern gene expression. Several methods have been developed to identify TFBSs in microalgal genomes. Experimental methods, such as electrophoretic mobility shift assay (EMSA) and chromatin immunoprecipitation, provide valuable insights into the direct interactions between transcription factors and their DNA binding sites (Galas and Schmitz, 1978; Garner and Revzin, 1981; Jothi et al., 2008; He et al., 2023). EMSAs involve incubating transcription factors with labeled DNA fragments containing potential TFBSs. The resulting shifts in mobility of the DNA fragments on a gel confirm the formation of transcription factor-DNA complexes and, therefore offer evidence of binding (Garner and Revzin, 1981). For instance, EMSA was used together with transcriptional correlation analyses and a yeast one-hybrid assay, to demonstrate that transcription factor CzMYB1 recognized the DNA sequence CNGTTA as its binding site (Shi M et al., 2022). This TFBS/TF pair is involved in the regulation of TAG accumulation in *Chromochloris zofingiensis*. EMSA was also used to determine whether the NobZIP77 TF interacts with the promoter of NoDGAT2B, which is involved in TAG synthesis in *Nannochloropsis oceanica* (Zhang et al., 2022).

Similarly, chromatin immunoprecipitation followed by sequencing (ChIP-Seq) can directly identify TFBSs by isolating DNA regions bound by *in vivo* interactions between transcription factors and DNA (Jothi et al., 2008; Lin et al., 2012; Veluchamy et al., 2015; Wei and Xu, 2018). For example, Ngan et al. (2015) used alterations in chromatin signatures to deduce the transcriptional regulators of the lipid biosynthesis pathway in *C. reinhardtii*, and one such TF gene was PSR1, which was validated via precise genetic manipulation (Ngan et al., 2015). Under nitrogen and sulfur starvation conditions, 694 genes were found to be involved in TAG accumulation and stress responses. Shen et al. (2021) also performed ChIP-Seq experiments: using the Motif Alignment and Search Tool, two motifs (TGTGTGTGTGTG and ACACACACACAC) were identified that would be of interest to conduct further research on to study the regulation mechanism of WRINKLED1 (WRI1) TF (Bailey et al., 2009; Shang et al., 2022). The WRI1 TF in *Arabidopsis thaliana* was shown to regulate many target genes involved in carbohydrate and lipid metabolism (Maeo et al., 2009; Shang et al., 2022).

Another experimental technique used to characterize TF/TFBS interactions is the protein binding microarray (PBM11), a method that contains all possible 11-mers to determine the DNA-binding specificities of TFs (Godoy et al., 2011). Using PBM11, Matthijs et al. (2017) analyzed how the TF bZIP14 regulates the tricarboxylic acid (TCA) cycle in the diatom *P. tricornutum* (Matthijs et al., 2017). This PBM11 analysis demonstrated that bZIP14 preferentially binds to motifs TGACGT and GTACGTA, both of which have an ACGT core. There are also other experimental methods developed to identify TFBSs, including systematic evolution of ligands by exponential enrichment (SELEX), DNA immunoprecipitation (DIP-chip), cleavage under targets and release using nucleases (CUT&RUN), and other techniques, which are tabled by Deng et al. (2022) (Liu et al., 2005; Chai et al., 2011; Jolma et al., 2015; Skene and Henikoff, 2017; Deng et al., 2022).

Alongside experimental methods, computational methods have also revolutionized the study of TFBS in microalgae, enabling large-scale analyses of potential TF/TFBS binding interactions. Databases like the Plant Transcription Factor Database (PlantTFDB) provide curated collections of known transcription factors, their binding motifs, and regulatory interactions based on experimental data, such as footprinting and ChIP-Seq (Jin et al., 2017). PlantTFDB curates and projects TFBS based on binding motifs derived from experiments from PlantCistromeDB, CIS-BP, JASPAR, UniPROBE, TRANSFAC, and MEME-ChIP (performs a comprehensive motif analysis under MEME-Suite) (Wingender et al., 1996; Matys et al., 2006; Weirauch et al., 2014; Bailey et al., 2015; Hume et al., 2015; O’Malley et al., 2016; Jin et al., 2017). Using some of these motif databases, Hu et al. (2014) predicted 78 interaction pairs between a TF and a TFBS motif that consisted of 34 TFs, 30 TFBS, and 950 target genes in *N. oceanica* IMET1 (Hu et al., 2014). The specificity of the predicted TFBSs was tested by comparing the TFBS motifs with verified motifs in TRANSFAC and PLACE (a database of nucleotide sequence motifs found in plant cis-acting regulatory DNA elements) by using STAMP (a web tool for investigating DNA-binding motif similarities) (Wingender et al., 1996; Higo et al., 1999, p. 199; Matys et al., 2006; Hu et al., 2014). Additionally, ChlamyNET is another database that provides a user interface to search for gene families using protein family identifiers and TFBS motifs in *Chlamydomonas* sp. (Romero-Campero et al., 2016). ChlamyNET predicts whether TFs might regulate other co-expressed genes and allows users to search for gene set enrichment analyses regarding gene ontology (GO) terms (Mochdia and Tamaki, 2021). Other TFBS databases and prediction methods include RegulonDB, SELEX_DB, GTRD, and other platforms that are also tabled by Deng et al. (2022) (Ponomarenko et al., 2000; Gama-Castro et al., 2016; Kolmykov et al., 2021; Deng et al., 2022). As can be seen, the aid of these motif databases enables researchers to predict TFBSs and confirm the regulatory importance of transcription factors.

### 3.3 Role of transcription factors in metabolic pathways

#### 3.3.1 Lipid biosynthesis

Microalgal lipids, especially TAG, are a promising feedstocks for biofuel production. The biosynthesis of microalgal lipids involves several fundamental biological processes, including carbon and nitrogen metabolism, energy metabolism, environmental stress response, and signaling regulation (Sun et al., 2018b). Microalgae exhibit the capacity to generate substantial quantities of lipids, with lipid content reaching more than 60% of the cell’s dry weight under some conditions (Morales et al., 2021). While notable advancements have been achieved in enhancing the production of algal lipids over the last decade, considerable further headway is necessary to scale up algal lipids and reach commodity pricing (Davis et al., 2011; Posewitz, 2017).

Genetic engineering focused on transcription factors has significantly progressed in enhancing lipid metabolism within various species of microalgae (Table 2; Bajhaiya et al., 2017; Bharadwaj et al., 2020; Kang et al., 2022; Muñoz et al., 2021; Sproles et al., 2021; Sun et al., 2018). In one approach, various studies have introduced plant transcription factors into microalgae. For instance, overexpressing *Dof* TFs from *Glycine Max* resulted in increased lipid yields in *C. reinhardtii* and *Chlorella ellipsoidea* (Ibáñez-Salazar et al., 2014; Zhang J et al., 2014; Salas-Montantes et al., 2018). Similarly, Leafy Cotyledon1 (LEC1) and AtWRI1 TFs from *Arabidopsis thaliana* were introduced into *Chlorella ellipsoidea* and *Nannochloropsis salina*, respectively, demonstrating enhanced lipid biosynthesis (Kang et al., 2017; Liu et al., 2021). In an alternative method, researchers have elevated the expression of native homologs of plant transcription factors engaged in microalgal lipid biosynthesis (Jia et al., 2022; Jia et al., 2019; Tokunaga et al., 2019). For example, a recent study showed that overexpressing the heat shock transcription factor PtHSF1 in *Phaeodactylum tricornutum* led to an increase in the synthesis of triacylglycerol and fucoxanthin (Song et al., 2023). Another set of techniques has identified microalgae transcription factors crucial for lipid accumulation. Ajjawi et al. (2017) and Thiriet-Rupert et al. (2018) employed RNA-Seq during nitrogen deprivation to identify transcription factors in *Nannochloropsis gaditana* and *Tisochrysis lutea*, respectively, that are involved in lipid metabolism (Ajjawi et al., 2017; Thiriet-Rupert et al., 2018). Takahashi et al. (2021) utilized existing transcriptome and phosphoproteome data to pinpoint potential transcription factors responsible for regulating TAG accumulation in the unicellular red alga *Cyanidioschyzon merolae* (Takahashi et al., 2021)*.* Likewise, Lv et al. (2013) performed transcriptome analysis to identify transcription factors that were up- or downregulated during the lipid accumulation process in *C. reinhardtii* (Lv et al., 2013).

**TABLE 2:** Comprehensive list of studies on transcription factor-based metabolic engineering in microalgae.

Microalgae species	Source species	Transcription factor	Experimental design	Stress condition	Biopathway	References
*Chlamydomonas reinhardtii*	*Chlamydomonas reinhardtii*	MYB1	Overexpression	Nitrogen Depletion	Lipid	Zhao et al. (2023)
*Chlamydomonas reinhardtii*	*Chlamydomonas reinhardtii*	MYB1	Overexpression	-	Starch	Zhao et al. (2023)
*Chlamydomonas reinhardtii*	*Chlamydomonas reinhardtii*	MYB1	Mutant	Nitrogen Depletion	Lipid	Choi et al. (2022), Shi Q et al. (2022)
*Chlamydomonas reinhardtii*	*Chlamydomonas reinhardtii*	NRR1	Mutant	Nitrogen Depletion	Lipid	Boyle et al. (2012)
*Chlamydomonas reinhardtii*	*Chlamydomonas reinhardtii*	PSR1	Mutant; Overexpression	Phosphorus Depletion	Starch	Bajhaiya et al. (2016)
*Chlamydomonas reinhardtii*	*Chlamydomonas reinhardtii*	PSR1	Mutant	Nitrogen Depletion	Lipid	Ngan et al. (2015)
*Chlamydomonas reinhardtii*	*Glycine max*	GmDof11	Overexpression	-	Lipid	Ibánez-Salazar et al. (2014)
*Chlamydomonas reinhardtii*	*Glycine max*	GmDof11	Overexpression	Nitrogen Depletion; Sulfur Depletion	Lipid	Salas-Montantes et al. (2018)
*Chlorella ellipsoidea*	*Glycine max*	GmDof4	Overexpression	-	Lipid	Zhang R et al. (2014)
*Chlamydomonas reinhardtii*	*Chlamydomonas reinhardtii*	CrDof	Overexpression	Heat Induction	Lipid	Jia et al. (2019)
*Chlamydomonas reinhardtii*	*Chlamydomonas reinhardtii*	CrDof	Overexpress DOF and knock down LACS2 and CIS lipid-related genes	Heat Shock; Nitrogen Depletion	Lipid	Jia et al. (2022)
*Chlorella vulgaris*	*Chlorella vulgaris*	CvDOF	Overexpression	Nitrogen Depletion	Lipid	Tokunaga et al. (2019)
*Chlamydomonas reinhardtii*	*Chlamydomonas reinhardtii*	CrbZIP1	Mutant	-	Lipid	Yamaoka et al. (2019)
*Chlamydomonas reinhardtii*	*Chlamydomonas reinhardtii*	CrbZIP2	Mutant	Nitrogen Depletion	Lipid	Bai et al. (2021)
*Chlamydomonas reinhardtii*	*Chlamydomonas reinhardtii*	LRL1	Mutant	Phosphorus Depletion	Lipid	Hidayati et al. (2019)
*Nannochloropsis gaditana*	*Nannochloropsis gaditana*	ZnCys	Mutant	Nitrogen Starvation	Lipid	Ajjawi et al. (2017)
*Nannochloropsis oceanica*	*Nannochloropsis oceanica*	NobZIP1	Overexpression	-	Lipid	Li et al. (2020)
*Chlamydomonas reinhardtii*	*Chlamydomonas reinhardtii*	CHT7	Mutation	Nitrogen Depletion	Lipid	Tsai et al. (2014)
*Nannochloropsis salina*	*Nannochloropsis salina*	NsbHLH2	Overexpression	Nitrogen Depletion; Osmotic Stress	Lipid	Kang et al. (2017)
*Chlorella ellipsoidea*	*Arabidopsis thaliana*	LEC1	Overexpression	-	Lipid	Liu et al. (2021)
*Nannochloropsis salina*	*Nannochloropsis salina*	NsbZIP1	Overexpression	Nitrogen Depletion; High Salt	Lipid	Kwon et al. (2018)
*Chlorella* sp. *HS2*	*Chlorella* sp. *HS2*	HSbZIP1	Overexpression	-	Lipid	Lee et al. (2023)
*Nannochloropsis salina*	*Arabidopsis thaliana*	AtWRI1	Overexpression	Nitrogen Depletion; High Salt	Lipid	Kang et al. (2017)
*Nannochloropsis oceanica*	*Nannochloropsis oceanica*	NO06G03670	Mutant	-	Lipid	Südfeld et al. (2021)
*Nannochloropsis oceanica*	*Nannochloropsis oceanica*	NO09G01030	Mutant	-	Lipid	Südfeld et al. (2021)
*Phaeodactylum tricornutum*	*Phaeodactylum tricornutum*	PtHSF1	Overexpression; Gene Silencing	-	Lipid	Song et al. (2023)
*Dunaliella parva*	*Dunaliella parva*	WRI1	Mutant	Nitrogen Depletion	Lipid	Shang et al. (2022)
*Chlamydomonas reinhardtii*	*Chlamydomonas reinhardtii*	Roc40	Mutant; Overexpression	Nitrogen Depletion	Lipid	Goncalves et al. (2016)
*Chromochloris zofingiensis*	*Chromochloris zofingiensis*	CzMYB1	-	Nitrogen Depletion	Lipid	Shi Q et al. (2022)
*Auxenochlorella protothecoides*	*Auxenochlorella protothecoides*	ApAP2, ApERF, ApMYB	-	Temperature Stress	Lipid	Xing G et al. (2021)
*Nannochloropsis oceanica*	*Nannochloropsis oceanica*	NobZIP77	Overexpression Knockout	Nitrogen Depletion and Light	Lipid	Zhang et al. (2022)
*Cyanidioschyzon merolae*	*Cyanidioschyzon merolae*	BRD1	Overexpression	Nitrogen Depletion	Lipid	Takahashi et al. (2021)
*Cyanidioschyzon merolae*	*Cyanidioschyzon merolae*	MYB3	Overexpression	Nitrogen Depletion	Lipid	Takahashi et al. (2021)
*Cyanidioschyzon merolae*	*Cyanidioschyzon merolae*	HSF1	Overexpression	Nitrogen Depletion	Lipid	Takahashi et al. (2021)
*Cyanidioschyzon merolae*	*Cyanidioschyzon merolae*	MYB4	Overexpression	Nitrogen Depletion	Lipid	Takahashi et al. (2021)

The table presents an exhaustive compilation of research studies centered on transcription factor-based metabolic engineering in microalgae. The source species column represents the species from which the transcription factor was sourced. Abbreviations: MYB, Myeloblastosis; NRR, Nitrogen Response Regulator; PSR, Phosphorus Starvation Response; Dof, DNA binding with One Finger; bZIP, Basic-region leucine zipper; LRR, Lipid Remodeling Regulator; ZnCys, Zn(II)_2_Cys_6_; CHT, Compromised Hydrolysis of Triacylglycerols; bHLH, basic helix-loop-helix; LEC, Leafy Cotyledon; WRI, WRINKLED1; HSF, Heat shock transcription factor; Roc, Rhythm of chloroplast; AP2, APETALA2; ERF, Ethylene Responsive Factor; BRD, Bromodomain-containing.

Potential regulatory pathways have been proposed for some of the TFs tabulated in Table 2. To elucidate the mechanism of underlying lipid production associated with specific TF expression, some studies have examined the genes potentially regulated by these TFs using experimental methods (Bajhaiya et al., 2016; Goncalves et al., 2016; Jia et al., 2022; Jia et al., 2019; Takahashi et al., 2021; Shang et al., 2022; Shi M et al., 2022). For example, Gargouri et al. (2015) combined omics (transcriptomic, proteomic, and metabolomic) analysis to identify transcriptional regulatory networks corresponding to oil accumulation under nitrogen deprivation in *C. reinhardtii* (Gargouri et al., 2015). Further, Xing G et al. (2021) analyzed the expression profile of 18 TFs and 32 lipid-metabolism-related genes to build a co-expression network to decipher the regulatory mechanism of lipid metabolism in *Auxenochlorella protothecoides* (Xing G et al., 2021). In their more recent research, Shi Q et al. (2022) conducted a comparison of TFs and differentially expressed genes (DEGs) related to lipid metabolism in *C. zofingiensis*, aiming to unveil potential TFs that may play a role (Shi M et al., 2022). Zheng et al. (2014) introduced the web-based AlgaePath database, which is tailored for *C. reinhardtii* and *Neodesmus* sp. UTEX 2219–4 strains. This resource provides information on genes, biological pathways, and NGS datasets, enabling pathway enrichment analysis for comparing transcript abundance among functionally related genes and supporting co-expression analysis (Zheng et al., 2014).

#### 3.3.2 Carbohydrate biosynthesis

Carbohydrates are energy and carbon-rich storage compounds found in most photosynthetic organisms, and starch (polysaccharide) is normally stored in the form of granules in the plastids of green microalgae, while in red algae, starch granules accumulate outside of the plastids (Busi et al., 2014). Starch degradation generally occurs during dark periods as a mechanism to provide energy to maintain cellular homeostasis, and starch metabolism also occurs in adverse environmental conditions to provide carbon for the biosynthesis of lipids (Johnson and Alric, 2013; Juergens et al., 2016). Most algal species contain about 30% starch content at the end of the day, but some strains such as *Dunaliella, Scenedesmus, Chlorella, Spirulina*, and *Chlamydomonas* can accumulate greater amounts of starch reaching more than 50% of their dry weight as starch (Hirano et al., 1997). Environmental stress, including nutrient stress, can also result in the increased accumulation of starch content, sometimes reaching as high as 60% (Yao et al., 2012; Shi Q et al., 2022).

Numerous research efforts in plants have focused on boosting starch accumulation via transcription factor engineering, either by overexpressing TFs from native or different species, or by knocking out other TF genes, although very few studies have been done on microalgae in this regard (López-González et al., 2019; Wu et al., 2019; Zhang et al., 2019; Fang et al., 2022). Bajhaiya et al. (2016), identified Phosphorus Starvation Response1 (PSR1) TF from *C. reinhardtii*, which regulates Phosphorus acquisition through the upregulation of phosphatases, and also results in higher expression of specific starch metabolism genes such as starch synthase (SSS1) and phosphorylases (SP1) (Table 2; Bajhaiya et al., 2016). They constructed a knockout mutant for this TF and found the inhibition of both lipid and starch accumulation under Phosphorus starvation conditions. They also generated PSR1 complementation lines in the *psr*1 strain and PSR1 overexpression lines to further understand the transcriptional regulation of lipid and starch metabolism. PSR1 overexpression lines regained their function and showed altered partitioning of carbon in the form of an increase in starch content and starch granules per cell, thus indicating higher starch metabolism and reduced content of neutral lipids. In a more recent study by Zhao et al. (2023), it was shown that overexpression of the MYB1 transcription factor in *C. reinhardtii* not only increased starch accumulation but also elevated the contents of lipids and proteins (Zhao et al., 2023).

In another study, researchers pinpointed TFs associated with the accumulation of twice the amount of storage lipids under nitrogen deprivation compared to the wild-type strain in a mutant strain of *T. lutea*. In this study, three of the identified TFs were found to be closely related to processes involving nitrogen and carbon recycling, ultimately contributing to carbohydrate synthesis (Thiriet-Rupert et al., 2018). In another study, multiomics analysis was done in *C. reinhardtii* under N deprivation condition to predict TFs and Transcriptional Repressors (TRs) for metabolic pathways through regulatory networks. They identified 241 putative TFs belonging to 37 different protein families and 173 putative TRs, which are members of 21 families based on the presence or absence of one or more DNA-binding domains. In carbohydrate metabolism they found Tab2 (RNA-binding protein) having high correlation with glycolytic enzyme transcripts, as well as G6P and F6P metabolites. PHD19 positively correlates with fructose, invertase (INV1), and alpha-amylase (AMA3) accumulation, whereas bZIP13 correlates with fructose, G1P, INV2, and phosphofructokinase (PFK2) but negatively correlates with three isoforms of glucose-1-phosphate adenyltransferase (GLGS1, GLGS2, and GLGS3), which catalyze the initial step in starch production. These results indicate that PHD19 and bZIP13 may be involved in regulating the switch from the gluconeogenic state to a glycolytic state, which occurs prior to the initiation of a lipid accumulation in *C. reinhardtii* during N deprivation. Other TFs and TRs involved in nitrogen metabolism, photosynthesis, photorespiration, chlorophyll metabolism, oxidative pentose phosphate pathway, citrate and glyoxylate cycle, amino acid metabolism, and lipid metabolism (Gargouri et al., 2015).

## 4 Challenges and future directions for engineering algae

To reach financial sustainability with algae-derived products, it is crucial to innovate and apply cultivation strategies that can produce biomass at rates above 30 g/m^2^ per day (US Department of energy multi-year program plan, 2014; Khan et al., 2018). This requirement highlights the need for advances across the entire production process to enable economical and widespread cultivation of algae in outdoor ponds (Rafa et al., 2021). Despite this, outdoor algae farming is vulnerable to events such as pond crashes, which can severely decrease biomass output (Klein and Davis, 2022; Molina-Grima et al., 2022; McGowen et al., 2023). Addressing these issues includes refining and employing tools for strains suitable for commercial production, and a thorough investigation of the regulatory roles of transcription factors in key biosynthetic pathways. The subsequent sections will delve deeper into the obstacles and potential developments in these areas.

### 4.1 Working with and genetically engineering extremophile microalgae

Extremophile microalgal strains are remarkable for their adaptability to grow under challenging environmental conditions. They can flourish in environments characterized by either acidic or alkaline pH (known as acidophiles and alkaliphiles), extreme pressure (barophiles), elevated light intensities, heightened CO_2_ concentrations, varying temperature extremes (both thermophilic and psychrophilic), high salinity levels (halophiles), and even in metal-rich surroundings (Rampelotto, 2013; Dalmaso et al., 2015; Zhu et al., 2020). Such strains have been identified and isolated from diverse, often inhospitable environments. An example includes *Chlamydomonas acidophila*, a green microalga adapted to acidic habitats, which was discovered in an acidic river in Spain (Cuaresma et al., 2011). Similarly, algae strains like *Chlorella protothecoides* var. *Acidicola* and *Euglena mutabilis* have been associated with abandoned copper mines in Spain and Wales (Ňancucheo and Barrie Johnson, 2012). The ability of these extremophiles to thrive in such harsh environments translates into several practical benefits. They inherently face lower contamination risks due to reduced competition for resources. Their robustness provides them with a unique resilience against climate fluctuations, making them particularly suitable for large-scale cultivation in open ponds, where they demonstrate a decreased vulnerability to environmental disruptions (Varshney et al., 2015). In addition to these cultivation benefits, extremophile strains hold immense biotechnological potential. They can generate an array of bioproducts, with commercial and therapeutic implications (Sydney et al., 2019). Varshney et al. (2015) have extensively documented extremophile microalgae strains and their potential for biotechnological applications (Varshney et al., 2015). For instance, species within the *Dunaliella* genus, notably high-salt-tolerant green marine microalgae, are proficient in producing products of commercial significance, ranging from carotenoids and polysaccharides to proteins, lipids, and vitamins (Moura et al., 2020). Specifically, *Dunaliella tertiolecta* is renowned for its lipid accumulation, suitable for biofuel production, and its synthesis of carotenoid pigments like β-Carotene, known for antioxidant, anticancer, and anti-inflammatory attributes (Ebadi et al., 2022). Astoundingly, some strains, dubbed as polyextremophiles, display tolerance to multiple adverse conditions simultaneously. *Cyanidioschyzon merolae* stands out as a polyextremophile resilient to both scorching temperatures and acidic environments. This unicellular red microalga produces bioactive compounds such as starch, β-glucan, β-carotene, zeaxanthin carotenoid pigments, and heat-stable phycocyanin (PC). These compounds find applications in diverse industries, including feed, cosmetics, nutrition, and biopharmaceuticals (Puzorjov et al., 2021; Villegas-Valencia et al., 2023).

While the prospects of using extremophilic microalgal strains in biotechnological endeavors are promising, there remains a need for more research. It is essential to either identify or engineer strains that can cater to the world’s escalating requirements (Varshney et al., 2015). Their intrinsic capacity to endure in an array of extreme conditions positions them as potential powerhouses for a thriving bioeconomy. However, to harness their full potential, the development and refinement of genetic tools, enhanced genetic engineering techniques, and comprehensive analysis of outdoor cultivation conditions are imperative.

### 4.2 Transcription factors and transcription factors binding sites in extremophile algae

Building on the potential of extremophilic microalgal strains for a thriving bioeconomy, the pursuit of a sustainable strategy necessitates a deeper exploration into their genomic landscape. Focusing on transcriptomic data analysis, prioritizing genome sequencing, and unraveling the regulatory networks of TFs linked to promising extremophiles is crucial for harnessing their full potential (Bajhaiya et al., 2017; Sydney et al., 2019). As we delve into understanding the intricacies of TFs regulatory networks in extremophilic microalgal strains, it is imperative to address challenges in identifying and confirming TFBSs. Experimental techniques such as DNase footprinting, EMSA, and yeast one-hybrid assays continue to lag behind the rapid accumulation of genome sequences (Hu et al., 2014). High-throughput experiments like ChIP-Seq are expensive and time-consuming. DNA affinity purification sequencing (DAP-seq) is another high-throughput method to classify TFBS by revealing the interactions between TFs and their motifs (O’Malley et al., 2016; Milito et al., 2023); however, DAP-seq can be laborious and expensive, hence bioinformatic prediction tools could pose as better alternatives (Hu et al., 2014; Shen et al., 2021; Milito et al., 2023). Computational approaches such as machine learning (ML) and deep learning (DL) have been recently developed and employed to determine TFBSs (Mochdia and Tamaki, 2021; Shen et al., 2021). In terms of ML, Artificial Neural Networks (ANNs) are becoming more prevalent in the biological sciences, especially Convolutional Neural Networks (CNNs), a type of ANN (Yang et al., 2020; Milito et al., 2023). Despite the increasing popularity of ML methods, there are still issues of high computational cost and challenges associated with interpreting the DNA sequence results (Milito et al., 2023). However, by embedding k-mer into CNNs, Shen et al. generated a robust prediction model named KEGRU to identify TFBS (Shen et al., 2018). There is currently a lack of research with explicit applications of such an integrative approach to microalgae, but these prediction models could significantly aid in the identification of TFBS.

As we explore the potential of an integrative approach in microalgal research, it is evident that current endeavors in modeling TF and TFBS relationships on a genome-wide scale are both recent and limited. The existing gap in research explicitly applying integrative approaches to microalgae presents an opportunity for computational prediction models to play a crucial role in unraveling TF-TFBS dynamics. However, this path is not without challenges, as the complex evolution of unicellular microorganisms and multicellular plants introduces uncertainties in modeling TF profiles across species, emphasizing the need for biochemically characterized binding motifs and TFs, and accurate algorithms in the computational prediction of the interactions of these elements and factors (Hu et al., 2014).

### 4.3 Lipids and carbohydrate biosynthesis

TFs play a pivotal role in modulating the expression of genes at various points within all biochemical pathways, either by enhancing or suppressing the abundance or activity of multiple essential enzymes. This is the driving force behind the growing interest among researchers in comprehending the functions of TFs (Courchesne et al., 2009; Bajhaiya et al., 2017). Numerous ongoing studies are investigating the impact of TFs in both plants and algae. However, there remains a considerable need for further research to uncover specific TFs and their associated target binding sites and associated genes in microalgae. The discovery of native TFs and their regulatory functions is at a nascent stage in algae, resulting in a notable gap in our understanding of comprehensive endogenous TF networks, particularly in non-model extremophile species. Bridging this knowledge gap will require the application of advanced techniques and computational tools. Additionally, these tools could play a crucial role in pinpointing homologs of plant transcription factors within microalgae, recognized for their capacity to enhance productivity. Multiomics methodologies, encompassing diverse analytical techniques such as genomics, epigenomics, transcriptomics, proteomics, and metabolomics, can be employed to elucidate the intricate regulatory networks linked to TFs involved in lipid or carbohydrate biosynthetic pathways (Figure 2; Gargouri et al., 2015; Liu and Benning, 2013). This holistic approach not only offers insights into the molecular intricacies of these pathways, but also serves as a powerful tool for discovering TFs and novel genes associated with TAG or starch accumulation. Recognizing that the genes regulated by specific TFs can exhibit variability across microalgae species, the wealth of genome-sequenced microalgae species opens up new avenues for uncovering key regulators. For instance, one study identified two distinct types of TFs, namely, heat-shock and bromodomain-containing TFs, as positive regulators of TAG accumulation in *C. merola*, although their precise mechanisms remain elusive, necessitating further research in the context of microalgae (Takahashi et al., 2021). As with many other organisms, microalgae have also displayed a close interplay between starch and lipid metabolism. Ongoing transcriptomics analyses are aimed at elucidating the regulatory mechanisms governing the partitioning of carbon between starch and lipid metabolism in extremophile microalgae (Sturme et al., 2018). In a separate study, researchers observed an upregulation of the DpWRI1 TF in *D. parva* under nitrogen deprivation. This TF regulates numerous target genes involved in carbohydrate metabolism, lipid metabolism, and photosynthesis to redirect carbon from starch to lipid accumulation (Shang et al., 2022). The precise metabolic nodes governing this carbon partitioning and their interconnected pathways remain a subject of ongoing investigation (Kareya et al., 2020; Sun et al., 2018).

## 5 Conclusion

Microalgae stand at the forefront of sustainable agriculture, heralding a new era where the constraints of traditional farming are circumvented through innovation. As the global population marches towards the 10 billion mark, the urgency for alternative resources intensifies. Microalgae’s rapid growth rates and versatility in non-traditional farming settings offer a sustainable and renewable lifeline for food, feed, and energy. They also play a critical role in carbon sequestration, aligning with environmental preservation efforts. However, economic factors present a paradox: large-scale, cost-effective production is needed for widespread application, yet such production depends on economic viability. This review has illuminated contemporary methods that enhance microalgae biomass quality, including gene editing and metabolic engineering. It also acknowledges the challenges ahead and underscores the importance of focusing on commercially viable strains. As research progresses, harnessing the full potential of microalgae requires not only scientific ingenuity but also a strategic approach to surmounting economic barriers, ensuring that microalgae can fulfill its promise as a scalable, low-cost solution for future generations.

## References

[B1] Acevedo-Rocha C. G. Gronenberg L. S. Mack M. Commichau F. M. Genee H. J. (2019). Microbial cell factories for the sustainable manufacturing of B vitamins. Curr. Opin. Biotechnol. 56, 18–29. 10.1016/j.copbio.2018.07.006 30138794

[B2] Ajjawi I. Verruto J. Aqui M. Soriaga L. B. Coppersmith J. Kwok K. (2017). Lipid production in Nannochloropsis gaditana is doubled by decreasing expression of a single transcriptional regulator. Nat. Biotechnol. 35, 647–652. 10.1038/nbt.3865 28628130

[B3] Antar M. Lyu D. Nazari M. Shah A. Zhou X. Smith D. L. (2021). Biomass for a sustainable bioeconomy: an overview of world biomass production and utilization. Renew. Sustain. Energy Rev. 139, 110691. 10.1016/j.rser.2020.110691

[B4] Antranikian G. Streit W. R. (2022). Microorganisms harbor keys to a circular bioeconomy making them useful tools in fighting plastic pollution and rising CO2 levels. Extremophiles 26, 10. 10.1007/s00792-022-01261-4 35118556 PMC8813813

[B5] Aratboni H. A. Rafiei N. Garcia-Granados R. Alemzadeh A. Morones-Ramírez J. R. (2019). Biomass and lipid induction strategies in microalgae for biofuel production and other applications. Microb. Cell Factories 18, 178. 10.1186/s12934-019-1228-4 PMC6805540 31638987

[B6] Araujo G. S. Matos L. J. B. L. Gonçalves L. R. B. Fernandes F. A. N. Farias W. R. L. (2011). Bioprospecting for oil producing microalgal strains: evaluation of oil and biomass production for ten microalgal strains. Bioresour. Technol. 102, 5248–5250. 10.1016/j.biortech.2011.01.089 21353534

[B7] Archer L. McGee D. Parkes R. Paskuliakova A. McCoy G. R. Adamo G. (2021). Antioxidant bioprospecting in microalgae: characterisation of the potential of two marine heterokonts from Irish waters. Appl. Biochem. Biotechnol. 193, 981–997. 10.1007/s12010-020-03467-8 33215392

[B8] Arnau J. Yaver D. Hjort C. M. (2019). “Strategies and challenges for the development of industrial enzymes using fungal cell factories,” in Grand challenges in fungal biotechnology. Grand challenges in biology and biotechnology (Cham: Springer), 179–210. 10.1007/978-3-030-29541-7_7

[B9] Arora N. Yen H.-W. Philippidis G. P. (2020). Harnessing the power of mutagenesis and adaptive laboratory evolution for high lipid production by oleaginous microalgae and yeasts. Sustainability 12, 5125. 10.3390/su12125125

[B10] Baek K. Kim D. H. Jeong J. Sim S. J. Melis A. Kim J.-S. (2016a). DNA-free two-gene knockout in Chlamydomonas reinhardtii via CRISPR-Cas9 ribonucleoproteins. Sci. Rep. 6, 30620. 10.1038/srep30620 27466170 PMC4964356

[B11] Baek K. Lee Y. Nam O. Park S. Sim S. J. Jin E. (2016b). Introducing Dunaliella LIP promoter containing light-inducible motifs improves transgenic expression in Chlamydomonas reinhardtii. Biotechnol. J. 11, 384–392. 10.1002/biot.201500269 26773277

[B12] Baeshen M. N. Al-Hejin A. M. Bora R. S. Ahmed M. M. M. Ramadan H. A. I. Saini K. S. (2015). Production of biopharmaceuticals in *E. coli*: current scenario and future perspectives. J. Microbiol. Biotechnol. 25, 953–962. 10.4014/jmb.1412.12079 25737124

[B13] Bailey T. L. Boden M. Buske F. A. Frith M. Grant C. E. Clementi L. (2009). MEME Suite: tools for motif discovery and searching. Nucleic Acids Res. 37, W202–W208. 10.1093/nar/gkp335 19458158 PMC2703892

[B14] Bailey T. L. Johnson J. Grant C. E. Noble W. S. (2015). The MEME suite. Nucleic Acids Res. 43, W39–W49. 10.1093/nar/gkv416 25953851 PMC4489269

[B15] Bajhaiya A. K. Dean A. P. Zeef L. A. H. Webster R. E. Pittman J. K. (2016). PSR1 is a global transcriptional regulator of Phosphorus deficiency responses and carbon storage metabolism in Chlamydomonas reinhardtii. Plant Physiol. 170, 1216–1234. 10.1104/pp.15.01907 26704642 PMC4775146

[B16] Bajhaiya A. K. Moreira J. Z. Pittman J. K. (2017). Transcriptional engineering of microalgae: prospects for high-value chemicals. Trends Biotechnol. 35, 95–99. 10.1016/j.tibtech.2016.06.001 27387061

[B17] Barbosa M. J. Janssen M. Südfeld C. D’Adamo S. Wijffels R. H. (2023). Hypes, hopes, and the way forward for microalgal biotechnology. Trends Biotechnol. 41, 452–471. 10.1016/j.tibtech.2022.12.017 36707271

[B18] Barclay W. Apt K. (2013). “Strategies for bioprospecting microalgae for potential commercial applications,” in Handbook of microalgal culture (United States: John Wiley and Sons, Ltd), 69–79. 10.1002/9781118567166.ch4

[B19] Barone P. W. Wiebe M. E. Leung J. C. Hussein I. T. M. Keumurian F. J. Bouressa J. (2020). Viral contamination in biologic manufacture and implications for emerging therapies. Nat. Biotechnol. 38, 563–572. 10.1038/s41587-020-0507-2 32341561

[B20] Bawa A. S. Anilakumar K. R. (2013). Genetically modified foods: safety, risks and public concerns—a review. J. Food Sci. Technol. 50, 1035–1046. 10.1007/s13197-012-0899-1 24426015 PMC3791249

[B21] Benedetti M. Vecchi V. Barera S. Dall’Osto L. (2018). Biomass from microalgae: the potential of domestication towards sustainable biofactories. Microb. Cell Factories 17, 173. 10.1186/s12934-018-1019-3 PMC6230293 30414618

[B22] Bharadwaj S. V. V. Ram S. Pancha I. Mishra S. (2020). “Chapter 14 - recent trends in strain improvement for production of biofuels from microalgae,” in Microalgae cultivation for biofuels production. Editor Yousuf A (Cambridge: Academic Press), 211–225. 10.1016/B978-0-12-817536-1.00014-X

[B23] Blount Z. D. (2015). The unexhausted potential of *E. coli* . eLife 4, e05826. 10.7554/eLife.05826 25807083 PMC4373459

[B24] Bohutskyi P. Liu K. Nasr L. K. Byers N. Rosenberg J. N. Oyler G. A. (2015). Bioprospecting of microalgae for integrated biomass production and phytoremediation of unsterilized wastewater and anaerobic digestion centrate. Appl. Microbiol. Biotechnol. 99, 6139–6154. 10.1007/s00253-015-6603-4 25947241

[B248] Boyle N. R. Dudley Page M. Liu B. Blaby I. K. Casero D. Kropat J. (2012). Three Acyltransferases and Nitrogen-responsive Regulator Are Implicated in Nitrogen Starvation-induced Triacylglycerol Accumulation in *Chlamydomonas* . J. Biol. Chem. 287 (19), 15811–15825. 10.1074/jbc.M111.334052 22403401 PMC3346115

[B25] Bugge M. M. Hansen T. Klitkou A. (2016). What is the bioeconomy? A review of the literature. Sustainability 8, 691. 10.3390/su8070691

[B26] Busi M. V. Barchiesi J. Martín M. Gomez-Casati D. F. (2014). Starch metabolism in green algae. Starch - Stärke 66, 28–40. 10.1002/star.201200211

[B27] Butelli E. Titta L. Giorgio M. Mock H.-P. Matros A. Peterek S. (2008). Enrichment of tomato fruit with health-promoting anthocyanins by expression of select transcription factors. Nat. Biotechnol. 26, 1301–1308. 10.1038/nbt.1506 18953354

[B28] Cai D. Zhu J. Zhu S. Lu Y. Zhang B. Lu K. (2019). Metabolic engineering of main transcription factors in carbon, nitrogen, and Phosphorus metabolisms for enhanced production of bacitracin in Bacillus licheniformis. ACS Synth. Biol. 8, 866–875. 10.1021/acssynbio.9b00005 30865822

[B29] Cerutti H. Johnson A. M. Gillham N. W. Boynton J. E. (1997). Epigenetic silencing of a foreign gene in nuclear transformants of Chlamydomonas. Plant Cell 9, 925–945. 10.1105/tpc.9.6.925 9212467 PMC156968

[B30] Chai C. Xie Z. Grotewold E. (2011). “SELEX (systematic evolution of ligands by EXponential enrichment), as a powerful tool for deciphering the protein–DNA interaction space,” in Plant transcription factors: methods and protocols, methods in molecular biology. Editors Yuan, L Perry S E (Totowa, NJ: Humana Press), 249–258. 10.1007/978-1-61779-154-3_14 21720957

[B31] Chen B. Wan C. Mehmood M. A. Chang J.-S. Bai F. Zhao X. (2017). Manipulating environmental stresses and stress tolerance of microalgae for enhanced production of lipids and value-added products–A review. Bioresour. Technol. SI:Algal Biorefinery 244, 1198–1206. 10.1016/j.biortech.2017.05.170 28601395

[B32] Chen C. Chen J. Wu G. Li L. Hu Z. Li X. (2023). A blue light-responsive strong synthetic promoter based on rational design in Chlamydomonas reinhardtii. Int. J. Mol. Sci. 24, 14596. 10.3390/ijms241914596 37834043 PMC10572394

[B33] Chen H. Wang Q. (2022). Microalgae-based green bio-manufacturing—how far from us. Front. Microbiol. 13, 832097. 10.3389/fmicb.2022.832097 35250947 PMC8891535

[B34] Chen X. Zhou L. Tian K. Kumar A. Singh S. Prior B. A. (2013). Metabolic engineering of *Escherichia coli*: a sustainable industrial platform for bio-based chemical production. Biotechnol. Adv. 31, 1200–1223. 10.1016/j.biotechadv.2013.02.009 23473968

[B35] Chettri D. Verma A. K. Verma A. K. (2023). Exploring the potential of microalgae cell factories for generation of biofuels. Biofuels 0, 1–13. 10.1080/17597269.2023.2233805

[B36] Cho J. S. Kim G. B. Eun H. Moon C. W. Lee S. Y. (2022). Designing microbial cell factories for the production of chemicals. JACS Au 2, 1781–1799. 10.1021/jacsau.2c00344 36032533 PMC9400054

[B37] Choi B. Y. Kim H. Shim D. Jang S. Yamaoka Y. Shin S. (2022). The Chlamydomonas bZIP transcription factor BLZ8 confers oxidative stress tolerance by inducing the carbon-concentrating mechanism. Plant Cell 34, 910–926. 10.1093/plcell/koab293 34893905 PMC8824676

[B38] Chu W.-L. (2017). Strategies to enhance production of microalgal biomass and lipids for biofuel feedstock. Eur. J. Phycol. 52, 419–437. 10.1080/09670262.2017.1379100

[B39] Corbu V. M. Gheorghe-Barbu I. Dumbravă A. Ș. Vrâncianu C. O. Șesan T. E. (2023). Current insights in fungal importance—a comprehensive review. Microorganisms 11, 1384. 10.3390/microorganisms11061384 37374886 PMC10304223

[B40] Corchero J. L. Gasser B. Resina D. Smith W. Parrilli E. Vázquez F. (2013). Unconventional microbial systems for the cost-efficient production of high-quality protein therapeutics. Biotechnol. Adv. 31, 140–153. 10.1016/j.biotechadv.2012.09.001 22985698

[B41] Courchesne N. M. D. Parisien A. Wang B. Lan C. Q. (2009). Enhancement of lipid production using biochemical, genetic and transcription factor engineering approaches. J. Biotechnol. 141, 31–41. 10.1016/j.jbiotec.2009.02.018 19428728

[B42] Cuaresma M. Casal C. Forján E. Vílchez C. (2011). Productivity and selective accumulation of carotenoids of the novel extremophile microalga Chlamydomonas acidophila grown with different carbon sources in batch systems. J. Ind. Microbiol. Biotechnol. 38, 167–177. 10.1007/s10295-010-0841-3 20811803

[B43] Dalmaso G. Z. L. Ferreira D. Vermelho A. B. (2015). Marine extremophiles: a source of hydrolases for biotechnological applications. Mar. Drugs 13, 1925–1965. 10.3390/md13041925 25854643 PMC4413194

[B44] Davis R. Aden A. Pienkos P. T. (2011). Techno-economic analysis of autotrophic microalgae for fuel production. Appl. Energy, Special Issue Energy algae Curr. status future trends 88, 3524–3531. 10.1016/j.apenergy.2011.04.018

[B45] De Bhowmick G. Koduru L. Sen R. (2015). Metabolic pathway engineering towards enhancing microalgal lipid biosynthesis for biofuel application—a review. Renew. Sustain. Energy Rev. 50, 1239–1253. 10.1016/j.rser.2015.04.131

[B46] Deng C. Wu Y. Lv X. Li J. Liu Y. Du G. (2022). Refactoring transcription factors for metabolic engineering. Biotechnol. Adv. 57, 107935. 10.1016/j.biotechadv.2022.107935 35271945

[B47] Dhankher O. P. Foyer C. H. (2018). Climate resilient crops for improving global food security and safety. Plant Cell Environ. 41, 877–884. 10.1111/pce.13207 29663504

[B48] Dhokane D. Shaikh A. Yadav A. Giri N. Bandyopadhyay A. Dasgupta S. (2023). CRISPR-based bioengineering in microalgae for production of industrially important biomolecules. Front. Bioeng. Biotechnol. 11, 1267826. 10.3389/fbioe.2023.1267826 37965048 PMC10641005

[B49] Diaz C. J. Douglas K. J. Kang K. Kolarik A. L. Malinovski R. Torres-Tiji Y. (2023). Developing algae as a sustainable food source. Front. Nutr. 9, 1029841. 10.3389/fnut.2022.1029841 36742010 PMC9892066

[B50] Dolganyuk V. Belova D. Babich O. Prosekov A. Ivanova S. Katserov D. (2020). Microalgae: a promising source of valuable bioproducts. Biomolecules 10, 1153. 10.3390/biom10081153 32781745 PMC7465300

[B51] Dumont J. Euwart D. Mei B. Estes S. Kshirsagar R. (2016). Human cell lines for biopharmaceutical manufacturing: history, status, and future perspectives. Crit. Rev. Biotechnol. 36, 1110–1122. 10.3109/07388551.2015.1084266 26383226 PMC5152558

[B52] Ebadi M. Mohammadi M. Pezeshki A. Jafari S. M. (2022). “Health benefits of beta-carotene,” in Handbook of food bioactive ingredients: properties and applications. Editors Jafari S. M Rashidinejad A Simal-Gandara J (Cham: Springer International Publishing), 1–26. 10.1007/978-3-030-81404-5_51-1

[B53] Einhaus A. Baier T. Rosenstengel M. Freudenberg R. A. Kruse O. (2021). Rational promoter engineering enables robust terpene production in microalgae. ACS Synth. Biol. 10, 847–856. 10.1021/acssynbio.0c00632 33764741

[B54] El Enshasy H. A. (2022). Fungal morphology: a challenge in bioprocess engineering industries for product development. Curr. Opin. Chem. Eng. 35, 100729. 10.1016/j.coche.2021.100729

[B55] El-Gendi H. Saleh A. K. Badierah R. Redwan E. M. El-Maradny Y. A. El-Fakharany E. M. (2021). A comprehensive insight into fungal enzymes: structure, classification, and their role in mankind’s challenges. J. Fungi 8, 23. 10.3390/jof8010023 PMC8778853 35049963

[B56] Fang W. Zhang Y. Zhang W. Gu J. Xiong F. An G. (2022). Rice transcription factor OsDOF18 enlarges the starch granule size by cytokinin. Curr. Plant Biol. 31, 100253. 10.1016/j.cpb.2022.100253

[B57] Ferrer-Miralles N. Villaverde A. (2013). Bacterial cell factories for recombinant protein production; expanding the catalogue. Microb. Cell Factories 12, 113. 10.1186/1475-2859-12-113 PMC3842683 24245806

[B58] Fields F. J. Ostrand J. T. Tran M. Mayfield S. P. (2019). Nuclear genome shuffling significantly increases production of chloroplast-based recombinant protein in Chlamydomonas reinhardtii. Algal Res. 41, 101523. 10.1016/j.algal.2019.101523

[B59] Fisher M. C. Gurr S. J. Cuomo C. A. Blehert D. S. Jin H. Stukenbrock E. H. (2020). Threats posed by the fungal kingdom to humans, wildlife, and agriculture. mBio 11, 00449–20. 10.1128/mBio.00449-20 PMC7403777 32371596

[B60] Fu W. Chaiboonchoe A. Khraiwesh B. Nelson D. R. Al-Khairy D. Mystikou A. (2016). Algal cell factories: approaches, applications, and potentials. Mar. Drugs 14, 225. 10.3390/md14120225 27983586 PMC5192462

[B61] Fu W. Guðmundsson Ó. Paglia G. Herjólfsson G. Andrésson Ó. S. Palsson B. Ø. (2013). Enhancement of carotenoid biosynthesis in the green microalga Dunaliella salina with light-emitting diodes and adaptive laboratory evolution. Appl. Microbiol. Biotechnol. 97, 2395–2403. 10.1007/s00253-012-4502-5 23095941 PMC3586100

[B62] Furtado A. Lupoi J. S. Hoang N. V. Healey A. Singh S. Simmons B. A. (2014). Modifying plants for biofuel and biomaterial production. Plant Biotechnol. J. 12, 1246–1258. 10.1111/pbi.12300 25431201

[B63] Galas D. J. Schmitz A. (1978). DNAase footprinting a simple method for the detection of protein-DNA binding specificity. Nucleic Acids Res. 5, 3157–3170. 10.1093/nar/5.9.3157 212715 PMC342238

[B64] Gama-Castro S. Salgado H. Santos-Zavaleta A. Ledezma-Tejeida D. Muñiz-Rascado L. García-Sotelo J. S. (2016). RegulonDB version 9.0: high-level integration of gene regulation, coexpression, motif clustering and beyond. Nucleic Acids Res. 44, D133–D143. 10.1093/nar/gkv1156 26527724 PMC4702833

[B65] Gargouri M. Park J.-J. Holguin F. O. Kim M.-J. Wang H. Deshpande R. R. (2015). Identification of regulatory network hubs that control lipid metabolism in Chlamydomonas reinhardtii. J. Exp. Bot. 66, 4551–4566. 10.1093/jxb/erv217 26022256 PMC4507760

[B66] Garner M. M. Revzin A. (1981). A gel electrophoresis method for quantifying the binding of proteins to specific DNA regions: application to components of the *Escherichia coli* lactose operon regulatory system. Nucleic Acids Res. 9, 3047–3060. 10.1093/nar/9.13.3047 6269071 PMC327330

[B67] Ghaderi D. Taylor R. E. Padler-Karavani V. Diaz S. Varki A. (2010). Implications of the presence of N-glycolylneuraminic acid in recombinant therapeutic glycoproteins. Nat. Biotechnol. 28, 863–867. 10.1038/nbt.1651 20657583 PMC3077421

[B68] Ghribi M. Nouemssi S. B. Meddeb-Mouelhi F. Desgagné-Penix I. (2020). Genome editing by CRISPR-cas: a game change in the genetic manipulation of Chlamydomonas. Life 10, 295. 10.3390/life10110295 33233548 PMC7699682

[B69] Godoy M. Franco-Zorrilla J. M. Pérez-Pérez J. Oliveros J. C. Lorenzo Ó. Solano R. (2011). Improved protein-binding microarrays for the identification of DNA-binding specificities of transcription factors. Plant J. 66, 700–711. 10.1111/j.1365-313X.2011.04519.x 21284757

[B70] Goncalves E. C. Koh J. Zhu N. Yoo M.-J. Chen S. Matsuo T. (2016). Nitrogen starvation-induced accumulation of triacylglycerol in the green algae: evidence for a role for ROC40, a transcription factor involved in circadian rhythm. Plant J. Cell Mol. Biol. 85, 743–757. 10.1111/tpj.13144 26920093

[B71] Gonçalves G. A. L. Bower D. M. Prazeres D. M. F. Monteiro G. A. Prather K. L. J. (2012). Rational engineering of *Escherichia coli* strains for plasmid biopharmaceutical manufacturing. Biotechnol. J. 7, 251–261. 10.1002/biot.201100062 21913330

[B72] Grama S. B. Liu Z. Li J. (2022). Emerging trends in genetic engineering of microalgae for commercial applications. Mar. Drugs 20, 285. 10.3390/md20050285 35621936 PMC9143385

[B73] Greiner A. Kelterborn S. Evers H. Kreimer G. Sizova I. Hegemann P. (2017). Targeting of photoreceptor genes in Chlamydomonas reinhardtii via zinc-finger nucleases and CRISPR/Cas9. Plant Cell 29, 2498–2518. 10.1105/tpc.17.00659 28978758 PMC5774583

[B74] Grigoriev I. V. Hayes R. D. Calhoun S. Kamel B. Wang A. Ahrendt S. (2021). PhycoCosm, a comparative algal genomics resource. Nucleic Acids Res. 49, D1004–D1011. 10.1093/nar/gkaa898 33104790 PMC7779022

[B75] Grubišić M. Šantek B. Zorić Z. Čošić Z. Vrana I. Gašparović B. (2022). Bioprospecting of microalgae isolated from the adriatic sea: characterization of biomass, pigment, lipid and fatty acid composition, and antioxidant and antimicrobial activity. Molecules 27, 1248. 10.3390/molecules27041248 35209036 PMC8875609

[B76] Guiry M. D. (2012). How many species of algae are there? J. Phycol. 48, 1057–1063. 10.1111/j.1529-8817.2012.01222.x 27011267

[B77] Guo A.-Y. Chen X. Gao G. Zhang H. Zhu Q.-H. Liu X.-C. (2008). PlantTFDB: a comprehensive plant transcription factor database. Nucleic Acids Res. 36, D966–D969. 10.1093/nar/gkm841 17933783 PMC2238823

[B78] Hankamer B. Pregelj L. O’Kane S. Hussey K. Hine D. (2023). Delivering impactful solutions for the bioeconomy. Trends Plant Sci. Spec. issue Food Secur. 28, 583–596. 10.1016/j.tplants.2023.02.007 36941134

[B79] Harlander S. K. (2002). The evolution of modern agriculture and its future with biotechnology. J. Am. Coll. Nutr. 21, 161S–165S. 10.1080/07315724.2002.10719260 12071299

[B80] He H. Yang M. Li S. Zhang G. Ding Z. Zhang L. (2023). Mechanisms and biotechnological applications of transcription factors. Synth. Syst. Biotechnol. 8, 565–577. 10.1016/j.synbio.2023.08.006 37691767 PMC10482752

[B251] Hidayati N. A. Yamada-Oshima Y. Iwai M. Yamano T. Kajikawa M. Sakurai N. (2019). Lipid remodeling regulator 1 (LRL1) is differently involved in the phosphorus-depletion response from PSR1 in Chlamydomonas reinhardtii. Plant J. 100 (03), 610–626. 10.1111/tpj.14473 31350858 PMC6899820

[B81] Higo K. Ugawa Y. Iwamoto M. Korenaga T. (1999). Plant cis-acting regulatory DNA elements (PLACE) database: 1999. Nucleic Acids Res. 27, 297–300. 10.1093/nar/27.1.297 9847208 PMC148163

[B82] Hirano A. Ueda R. Hirayama S. Ogushi Y. (1997). CO2 fixation and ethanol production with microalgal photosynthesis and intracellular anaerobic fermentation. Energy 22, 137–142. 10.1016/S0360-5442(96)00123-5

[B83] Hu J. Wang D. Chen H. Wang Q. (2023). Advances in genetic engineering in improving photosynthesis and microalgal productivity. Int. J. Mol. Sci. 24, 1898. 10.3390/ijms24031898 36768215 PMC9915242

[B84] Hu J. Wang D. Li J. Jing G. Ning K. Xu J. (2014). Genome-wide identification of transcription factors and transcription-factor binding sites in oleaginous microalgae Nannochloropsis. Sci. Rep. 4, 5454. 10.1038/srep05454 24965723 PMC5154493

[B85] Hume M. A. Barrera L. A. Gisselbrecht S. S. Bulyk M. L. (2015). UniPROBE, update 2015: new tools and content for the online database of protein-binding microarray data on protein–DNA interactions. Nucleic Acids Res. 43, D117–D122. 10.1093/nar/gku1045 25378322 PMC4383892

[B86] Ibáñez-Salazar A. Rosales-Mendoza S. Rocha-Uribe A. Ramírez-Alonso J. I. Lara-Hernández I. Hernández-Torres A. (2014). Over-expression of Dof-type transcription factor increases lipid production in Chlamydomonas reinhardtii. J. Biotechnol. 184, 27–38. 10.1016/j.jbiotec.2014.05.003 24844864

[B87] Jeong B. Jang J. Jin E. (2023). Genome engineering via gene editing technologies in microalgae. Bioresour. Technol. 373, 128701. 10.1016/j.biortech.2023.128701 36746216

[B88] Jia B. Xie X. Wu M. Lin Z. Yin J. lou S. (2019). Understanding the functions of endogenous DOF transcript factor in Chlamydomonas reinhardtii. Biotechnol. Biofuels 12, 67. 10.1186/s13068-019-1403-1 30972144 PMC6436238

[B89] Jia B. Yin J. Li X. Li Y. Yang X. Lan C. (2022). Increased lipids in Chlamydomonas reinhardtii by multiple regulations of DOF, LACS2, and CIS1. Int. J. Mol. Sci. 23, 10176. 10.3390/ijms231710176 36077572 PMC9456367

[B90] Jia Y.-L. Li J. Nong F.-T. Yan C.-X. Ma W. Zhu X.-F. (2023). Application of adaptive laboratory evolution in lipid and terpenoid production in yeast and microalgae. ACS Synth. Biol. 12, 1396–1407. 10.1021/acssynbio.3c00179 37084707

[B91] Jiang W. Brueggeman A. J. Horken K. M. Plucinak T. M. Weeks D. P. (2014). Successful transient expression of Cas9 and single guide RNA genes in Chlamydomonas reinhardtii. Eukaryot. Cell 13, 1465–1469. 10.1128/ec.00213-14 25239977 PMC4248704

[B92] Jin J. Tian F. Yang D.-C. Meng Y.-Q. Kong L. Luo J. (2017). PlantTFDB 4.0: toward a central hub for transcription factors and regulatory interactions in plants. Nucleic Acids Res. 45, D1040–D1045. 10.1093/nar/gkw982 27924042 PMC5210657

[B93] Jinkerson R. E. Jonikas M. C. (2015). Molecular techniques to interrogate and edit the Chlamydomonas nuclear genome. Plant J. 82, 393–412. 10.1111/tpj.12801 25704665

[B94] Jo C. Zhang J. Tam J. M. Church G. M. Khalil A. S. Segrè D. (2023). Unlocking the magic in mycelium: using synthetic biology to optimize filamentous fungi for biomanufacturing and sustainability. Mat. Today Bio 19, 100560. 10.1016/j.mtbio.2023.100560 PMC9900623 36756210

[B95] Johnson X. Alric J. (2013). Central carbon metabolism and electron transport in Chlamydomonas reinhardtii: metabolic constraints for carbon partitioning between oil and starch. Eukaryot. Cell 12, 776–793. 10.1128/EC.00318-12 23543671 PMC3675994

[B96] Jolma A. Yin Y. Nitta K. R. Dave K. Popov A. Taipale M. (2015). DNA-dependent formation of transcription factor pairs alters their binding specificity. Nature 527, 384–388. 10.1038/nature15518 26550823

[B97] Jothi R. Cuddapah S. Barski A. Cui K. Zhao K. (2008). Genome-wide identification of *in vivo* protein-DNA binding sites from ChIP-Seq data. Nucleic Acids Res. 36, 5221–5231. 10.1093/nar/gkn488 18684996 PMC2532738

[B98] Juergens M. T. Disbrow B. Shachar-Hill Y. (2016). The relationship of triacylglycerol and starch accumulation to carbon and energy flows during nutrient deprivation in Chlamydomonas reinhardtii. Plant Physiol. 171, 2445–2457. 10.1104/pp.16.00761 27325664 PMC4972295

[B99] Kamaludin N. H. Feisal N. A. S. (2023). “Biomanufacturing for sustainable production of biomolecules: Pseudomonas putida cell factory,” in Biomanufacturing for sustainable production of biomolecules. Editors Singh, V Show P. L (Singapore: Springer Nature), 175–188. 10.1007/978-981-19-7911-8_9

[B100] Kang N. K. Baek K. Koh H. G. Atkinson C. A. Ort D. R. Jin Y.-S. (2022). Microalgal metabolic engineering strategies for the production of fuels and chemicals. Bioresour. Technol. 345, 126529. 10.1016/j.biortech.2021.126529 34896527

[B101] Kang N. K. Kim E. K. Kim Y. U. Lee B. Jeong W.-J. Jeong B.-R. (2017). Increased lipid production by heterologous expression of AtWRI1 transcription factor in Nannochloropsis salina. Biotechnol. Biofuels 10, 231. 10.1186/s13068-017-0919-5 29046718 PMC5635583

[B102] Kareya M. S. Mariam I. Shaikh K. M. Nesamma A. A. Jutur P. P. (2020). Photosynthetic carbon partitioning and metabolic regulation in response to very-low and high CO2 in microchloropsis gaditana NIES 2587. Front. Plant Sci. 11, 981. 10.3389/fpls.2020.00981 32719702 PMC7348049

[B103] Kelterborn S. Boehning F. Sizova I. Baidukova O. Evers H. Hegemann P. (2022). “Gene editing in green alga Chlamydomonas reinhardtii via CRISPR-cas9 ribonucleoproteins,” in Plant synthetic biology: methods and protocols, methods in molecular biology. Editor Zurbriggen M. D (New York, NY: Springer US), 45–65. 10.1007/978-1-0716-1791-5_3 35188655

[B104] Khan M. I. Shin J. H. Kim J. D. (2018). The promising future of microalgae: current status, challenges, and optimization of a sustainable and renewable industry for biofuels, feed, and other products. Microb. Cell Factories 17, 36. 10.1186/s12934-018-0879-x PMC5836383 29506528

[B105] Khoo K. S. Ahmad I. Chew K. W. Iwamoto K. Bhatnagar A. Show P. L. (2023). Enhanced microalgal lipid production for biofuel using different strategies including genetic modification of microalgae: a review. Prog. Energy Combust. Sci. 96, 101071. 10.1016/j.pecs.2023.101071

[B106] Khoo K. S. Chew K. W. Yew G. Y. Leong W. H. Chai Y. H. Show P. L. (2020). Recent advances in downstream processing of microalgae lipid recovery for biofuel production. Bioresour. Technol. 304, 122996. 10.1016/j.biortech.2020.122996 32115347

[B107] Kim J. Lee S. Baek K. Jin E. (2020). Site-specific gene knock-out and on-site heterologous gene overexpression in Chlamydomonas reinhardtii via a CRISPR-cas9-mediated knock-in method. Front. Plant Sci. 11, 306. 10.3389/fpls.2020.00306 32265959 PMC7099044

[B108] Klein B. Davis R. (2022). Algal biomass production via open pond algae farm cultivation: 2022 state of technology and future research. United States: Renewable Energy Laboratory.

[B109] Koh L. P. Ghazoul J. (2008). Biofuels, biodiversity, and people: understanding the conflicts and finding opportunities. Biol. Conserv. 141, 2450–2460. 10.1016/j.biocon.2008.08.005

[B110] Kolmykov S. Yevshin I. Kulyashov M. Sharipov R. Kondrakhin Y. Makeev V. J. (2021). GTRD: an integrated view of transcription regulation. Nucleic Acids Res. 49, D104–D111. 10.1093/nar/gkaa1057 33231677 PMC7778956

[B111] Koppolu V. Vasigala V. K. (2016). Role of *Escherichia coli* in biofuel production. Microbiol. Insights 9, MBI.S10878. 10.4137/MBI.S10878 PMC4946582 27441002

[B112] Kumar G. Shekh A. Jakhu S. Sharma Y. Kapoor R. Sharma T. R. (2020). Bioengineering of microalgae: recent advances, perspectives, and regulatory challenges for industrial application. Front. Bioeng. Biotechnol. 8, 914. 10.3389/fbioe.2020.00914 33014997 PMC7494788

[B113] Kumar V. Sharma N. Jaiswal K. K. Vlaskin M. S. Nanda M. Tripathi M. K. (2021). Microalgae with a truncated light-harvesting antenna to maximize photosynthetic efficiency and biomass productivity: recent advances and current challenges. Process Biochem. 104, 83–91. 10.1016/j.procbio.2021.03.006

[B114] Kurukulasuriya P. Rosenthal S. (2013). Climate change and agriculture: a review of impacts and adaptations. Open Knowledge Repository beta.

[B115] Kwon S. Kang N. K. Koh H. G. Shin S.-E. Lee B. Jeong B. (2018). Enhancement of biomass and lipid productivity by overexpression of a bZIP transcription factor in Nannochloropsis salina. Biotechnol. Bioeng. 115, 331–340. 10.1002/bit.26465 28976541

[B116] Lam T. P. Lee T.-M. Chen C.-Y. Chang J.-S. (2018). Strategies to control biological contaminants during microalgal cultivation in open ponds. Bioresour. Technol. 252, 180–187. 10.1016/j.biortech.2017.12.088 29306613

[B117] Lane T. W. (2022). Barriers to microalgal mass cultivation. Curr. Opin. Biotechnol. 73, 323–328. 10.1016/j.copbio.2021.09.013 34710649

[B118] Lang D. Weiche B. Timmerhaus G. Richardt S. Riaño-Pachón D. M. Corrêa L. G. G. (2010). Genome-wide phylogenetic comparative analysis of plant transcriptional regulation: a timeline of loss, gain, expansion, and correlation with complexity. Genome Biol. Evol. 2, 488–503. 10.1093/gbe/evq032 20644220 PMC2997552

[B119] LaPanse A. J. Krishnan A. Posewitz M. C. (2021). Adaptive Laboratory Evolution for algal strain improvement: methodologies and applications. Algal Res. 53, 102122. 10.1016/j.algal.2020.102122

[B120] Larkum A. W. D. Ross I. L. Kruse O. Hankamer B. (2012). Selection, breeding and engineering of microalgae for bioenergy and biofuel production. Trends Biotechnol. 30, 198–205. 10.1016/j.tibtech.2011.11.003 22178650

[B121] Lee T.-M. Lin J.-Y. Tsai T.-H. Yang R.-Y. Ng I.-S. (2023). Clustered regularly interspaced short palindromic repeats (CRISPR) technology and genetic engineering strategies for microalgae towards carbon neutrality: a critical review. Bioresour. Technol. 368, 128350. 10.1016/j.biortech.2022.128350 36414139

[B122] Li F. Vijayasankaran N. Shen A. (Yijuan) Kiss R. Amanullah A. (2010). Cell culture processes for monoclonal antibody production. mAbs 2, 466–479. 10.4161/mabs.2.5.12720 20622510 PMC2958569

[B123] Li M. Xu J. Gao Z. Tian H. Gao Y. Kariman K. (2020). Genetically modified crops are superior in their nitrogen use efficiency-A meta-analysis of three major cereals. Sci. Rep. 10, 8568. 10.1038/s41598-020-65684-9 32444783 PMC7244766

[B124] Liang L. Liu R. Freed E. F. Eckert C. A. (2020). Synthetic biology and metabolic engineering employing *Escherichia coli* for C2–C6 bioalcohol production. Front. Bioeng. Biotechnol. 8, 710. 10.3389/fbioe.2020.00710 32719784 PMC7347752

[B125] Lin J.-Y. Lin W.-R. Ng I.-S. (2021). CRISPRa/i with Adaptive Single Guide Assisted Regulation DNA (ASGARD) mediated control of Chlorella sorokiniana to enhance lipid and protein production. Biotechnol. J. 17, 2100514. 10.1002/biot.202100514 34800080

[B126] Lin W.-R. Tan S.-I. Hsiang C.-C. Sung P.-K. Ng I.-S. (2019). Challenges and opportunity of recent genome editing and multi-omics in cyanobacteria and microalgae for biorefinery. Bioresour. Technol. 291, 121932. 10.1016/j.biortech.2019.121932 31387837

[B127] Lin X. Tirichine L. Bowler C. (2012). Protocol: chromatin immunoprecipitation (ChIP) methodology to investigate histone modifications in two model diatom species. Plant Methods 8, 48. 10.1186/1746-4811-8-48 23217141 PMC3546051

[B128] Liu B. Benning C. (2013). Lipid metabolism in microalgae distinguishes itself. Curr. Opin. Biotechnol. Food Biotechnol. • Plant Biotechnol. 24, 300–309. 10.1016/j.copbio.2012.08.008 22981869

[B129] Liu J. Fernie A. R. Yan J. (2020). The past, present, and future of maize improvement: domestication, genomics, and functional genomic routes toward crop enhancement. Plant Commun. 1, 100010. 10.1016/j.xplc.2019.100010 33404535 PMC7747985

[B130] Liu X. Noll D. M. Lieb J. D. Clarke N. D. (2005). DIP-chip: rapid and accurate determination of DNA-binding specificity. Genome Res. 15, 421–427. 10.1101/gr.3256505 15710749 PMC551568

[B131] Liu X. Zhang D. Zhang J. Chen Y. Liu X. Fan C. (2021). Overexpression of the transcription factor AtLEC1 significantly improved the lipid content of Chlorella ellipsoidea. Front. Bioeng. Biotechnol. 9, 626162. 10.3389/fbioe.2021.626162 33681161 PMC7925920

[B132] Liu Y. Liu L. Shin H. Chen R. R. Li J. Du G. (2013). Pathway engineering of Bacillus subtilis for microbial production of N-acetylglucosamine. Metab. Eng. 19, 107–115. 10.1016/j.ymben.2013.07.002 23876412

[B133] López-González C. Juárez-Colunga S. Morales-Elías N. C. Tiessen A. (2019). Exploring regulatory networks in plants: transcription factors of starch metabolism. PeerJ 7, e6841. 10.7717/peerj.6841 31328026 PMC6625501

[B134] Lübeck M. Lübeck P. S. (2022). Fungal cell factories for efficient and sustainable production of proteins and peptides. Microorganisms 10, 753. 10.3390/microorganisms10040753 35456803 PMC9025306

[B135] Lv H. Qu G. Qi X. Lu L. Tian C. Ma Y. (2013). Transcriptome analysis of Chlamydomonas reinhardtii during the process of lipid accumulation. Genomics 101, 229–237. 10.1016/j.ygeno.2013.01.004 23396177

[B136] Maeo K. Tokuda T. Ayame A. Mitsui N. Kawai T. Tsukagoshi H. (2009). An AP2-type transcription factor, WRINKLED1, of *Arabidopsis thaliana* binds to the AW-box sequence conserved among proximal upstream regions of genes involved in fatty acid synthesis. Plant J. 60, 476–487. 10.1111/j.1365-313X.2009.03967.x 19594710

[B137] Maity K. G. (2019). “Tridib kumar Bhowmick,” in Sustainable downstream processing of microalgae for industrial application. Editor Kumar S (Boca Raton: CRC Press). 10.1201/9780429027970

[B138] Maity S. Mallick N. (2022). Bioprospecting marine microalgae and cyanobacteria as alternative feedstocks for bioethanol production. Sustain. Chem. Pharm. 29, 100798. 10.1016/j.scp.2022.100798

[B139] Manuell A. L. Beligni M. V. Elder J. H. Siefker D. T. Tran M. Weber A. (2007). Robust expression of a bioactive mammalian protein in Chlamydomonas chloroplast. Plant Biotechnol. J. 5, 402–412. 10.1111/j.1467-7652.2007.00249.x 17359495

[B140] Maréchal E. (2021). Grand challenges in microalgae domestication. Front. Plant Sci. 12, 764573. 10.3389/fpls.2021.764573 34630500 PMC8495258

[B141] Matthijs M. Fabris M. Obata T. Foubert I. Franco-Zorrilla J. M. Solano R. (2017). The transcription factor bZIP14 regulates the TCA cycle in the diatom Phaeodactylum tricornutum. EMBO J. 36, 1559–1576. 10.15252/embj.201696392 28420744 PMC5452028

[B142] Matys V. Kel-Margoulis O. V. Fricke E. Liebich I. Land S. Barre-Dirrie A. (2006). TRANSFAC(R) and its module TRANSCompel(R): transcriptional gene regulation in eukaryotes. Nucleic Acids Res. 34, D108–D110. 10.1093/nar/gkj143 16381825 PMC1347505

[B143] McGowen J. Knoshaug E. P. Laurens L. M. L. Forrester J. (2023). Outdoor annual algae productivity improvements at the pre-pilot scale through crop rotation and pond operational management strategies. Algal Res. 70, 102995. 10.1016/j.algal.2023.102995

[B144] McQuillan J. L. Berndt A. J. Sproles A. E. Mayfield S. P. Pandhal J. (2022). Novel cis-regulatory elements as synthetic promoters to drive recombinant protein expression from the Chlamydomonas reinhardtii nuclear genome. New Biotechnol. 68, 9–18. 10.1016/j.nbt.2022.01.001 34990855

[B145] Meyer V. Andersen M. R. Brakhage A. A. Braus G. H. Caddick M. X. Cairns T. C. (2016). Current challenges of research on filamentous fungi in relation to human welfare and a sustainable bio-economy: a white paper. Fungal Biol. Biotechnol. 3, 6. 10.1186/s40694-016-0024-8 28955465 PMC5611618

[B146] Milito A. Aschern M. McQuillan J. L. Yang J.-S. (2023). Challenges and advances towards the rational design of microalgal synthetic promoters in Chlamydomonas reinhardtii. J. Exp. Bot. 74, 3833–3850. 10.1093/jxb/erad100 37025006

[B147] Mochdia K. Tamaki S. (2021). Transcription factor-based genetic engineering in microalgae. Plants 10, 1602. 10.3390/plants10081602 34451646 PMC8399792

[B148] Molina-Grima E. García-Camacho F. Acién-Fernández F. G. Sánchez-Mirón A. Plouviez M. Shene C. (2022). Pathogens and predators impacting commercial production of microalgae and cyanobacteria. Biotechnol. Adv. 55, 107884. 10.1016/j.biotechadv.2021.107884 34896169

[B149] Morales M. Aflalo C. Bernard O. (2021). Microalgal lipids: a review of lipids potential and quantification for 95 phytoplankton species. Biomass Bioenergy 150, 106108. 10.1016/j.biombioe.2021.106108

[B150] Moura Y. A. S. Viana-Marques D. A. Porto A. L. F. Bezerra R. P. Converti A. (2020). Pigments production, growth kinetics, and bioenergetic patterns in Dunaliella tertiolecta (Chlorophyta) in response to different culture media. Energies 13, 5347. 10.3390/en13205347

[B151] Munir N. Hasnain M. Roessner U. Abideen Z. (2022). Strategies in improving plant salinity resistance and use of salinity resistant plants for economic sustainability. Crit. Rev. Environ. Sci. Technol. 52, 2150–2196. 10.1080/10643389.2021.1877033

[B152] Muñoz C. F. Südfeld C. Naduthodi M. I. S. Weusthuis R. A. Barbosa M. J. Wijffels R. H. (2021). Genetic engineering of microalgae for enhanced lipid production. Biotechnol. Adv. 52, 107836. 10.1016/j.biotechadv.2021.107836 34534633

[B153] Naduthodi M. I. S. Mohanraju P. Südfeld C. D’Adamo S. Barbosa M. J. van der Oost J. (2019). CRISPR–Cas ribonucleoprotein mediated homology-directed repair for efficient targeted genome editing in microalgae Nannochloropsis oceanica IMET1. Biotechnol. Biofuels 12, 66. 10.1186/s13068-019-1401-3 30962821 PMC6432748

[B154] Ňancucheo I. Barrie Johnson D. (2012). Acidophilic algae isolated from mine-impacted environments and their roles in sustaining heterotrophic acidophiles. Front. Microbiol. 3, 325. 10.3389/fmicb.2012.00325 22973267 PMC3438993

[B155] Naranjo‐Ortiz M. A. Gabaldón T. (2020). Fungal evolution: cellular, genomic and metabolic complexity. Biol. Rev. Camb. Philos. Soc. 95, 1198–1232. 10.1111/brv.12605 32301582 PMC7539958

[B156] Navarrete C. Martínez J. L. (2020). Non-conventional yeasts as superior production platforms for sustainable fermentation based bio-manufacturing processes. Aims Bioeng. 7, 289–305. 10.3934/bioeng.2020024

[B157] Nayak M. Suh W. I. Oh Y. T. Ryu A. J. Jeong K. J. Kim M. (2022). Directed evolution of Chlorella sp. HS2 towards enhanced lipid accumulation by ethyl methanesulfonate mutagenesis in conjunction with fluorescence-activated cell sorting based screening. Fuel 316, 123410. 10.1016/j.fuel.2022.123410

[B158] Neofotis P. Huang A. Sury K. Chang W. Joseph F. Gabr A. (2016). Characterization and classification of highly productive microalgae strains discovered for biofuel and bioproduct generation. Algal Res. 15, 164–178. 10.1016/j.algal.2016.01.007

[B159] Neupert J. Gallaher S. D. Lu Y. Strenkert D. Segal N. Barahimipour R. (2020). An epigenetic gene silencing pathway selectively acting on transgenic DNA in the green alga Chlamydomonas. Nat. Commun. 11, 6269. 10.1038/s41467-020-19983-4 33293544 PMC7722844

[B160] Ng I.-S. Tan S.-I. Kao P.-H. Chang Y.-K. Chang J.-S. (2017). Recent developments on genetic engineering of microalgae for biofuels and bio-based chemicals. Biotechnol. J. 12, 1600644. 10.1002/biot.201600644 28786539

[B161] Ngan C. Y. Wong C.-H. Choi C. Yoshinaga Y. Louie K. Jia J. (2015). Lineage-specific chromatin signatures reveal a regulator of lipid metabolism in microalgae. Nat. Plants 1, 15107–15112. 10.1038/nplants.2015.107 27250540

[B162] Nguyen A. D. Lee E. Y. (2021). Engineered methanotrophy: a sustainable solution for methane-based industrial biomanufacturing. Trends Biotechnol. 39, 381–396. 10.1016/j.tibtech.2020.07.007 32828555

[B163] Nur M. M. A. Buma A. G. J. (2019). Opportunities and challenges of microalgal cultivation on wastewater, with special focus on palm oil mill effluent and the production of high value compounds. Waste Biomass Valorization 10, 2079–2097. 10.1007/s12649-018-0256-3

[B164] O’Malley R. C. Huang S.-S. C. Song L. Lewsey M. G. Bartlett A. Nery J. R. (2016). Cistrome and epicistrome features shape the regulatory DNA landscape. Cell 165, 1280–1292. 10.1016/j.cell.2016.04.038 27203113 PMC4907330

[B165] Onyeaka H. Miri T. Obileke K. Hart A. Anumudu C. Al-Sharify Z. T. (2021). Minimizing carbon footprint via microalgae as a biological capture. Carbon Capture Sci. Technol. 1, 100007. 10.1016/j.ccst.2021.100007

[B166] Patel A. K. Singhania R. R. Pandey A. (2016). Novel enzymatic processes applied to the food industry. Curr. Opin. 7, 64–72. 10.1016/j.cofs.2015.12.002

[B167] Patel V. K. Das A. Kumari R. Kajla S. (2023). Recent progress and challenges in CRISPR-Cas9 engineered algae and cyanobacteria. Algal Res. 71, 103068. 10.1016/j.algal.2023.103068

[B168] Patel V. K. Soni N. Prasad V. Sapre A. Dasgupta S. Bhadra B. (2019). CRISPR–Cas9 system for genome engineering of photosynthetic microalgae. Mol. Biotechnol. 61, 541–561. 10.1007/s12033-019-00185-3 31140149

[B169] Ponomarenko J. V. Orlova G. V. Ponomarenko M. P. Lavryushev S. V. Frolov A. S. Zybova S. V. (2000). SELEX_DB: an activated database on selected randomized DNA/RNA sequences addressed to genomic sequence annotation. Nucleic Acids Res. 28, 205–208. 10.1093/nar/28.1.205 10592226 PMC102392

[B170] Posewitz M. C. (2017). Algal oil productivity gets a fat bonus. Nat. Biotechnol. 35, 636–638. 10.1038/nbt.3920 28700558

[B171] Puzorjov A. Dunn K. E. McCormick A. J. (2021). Production of thermostable phycocyanin in a mesophilic cyanobacterium. Metab. Eng. Commun. 13, e00175. 10.1016/j.mec.2021.e00175 34168957 PMC8209669

[B172] Rafa N. Ahmed S. F. Badruddin I. A. Mofijur M. Kamangar S. (2021). Strategies to produce cost-effective third-generation biofuel from microalgae. Front. Energy Res. 9. 10.3389/fenrg.2021.749968

[B173] Ramanna L. Rawat I. Bux F. (2017). Light enhancement strategies improve microalgal biomass productivity. Renew. Sustain. Energy Rev. 80, 765–773. 10.1016/j.rser.2017.05.202

[B174] Ramos J. Valdivia M. García‐Lorente F. Segura A. (2016). Benefits and perspectives on the use of biofuels. Microb. Biotechnol. 9, 436–440. 10.1111/1751-7915.12356 27115937 PMC4919985

[B175] Rampelotto P. H. (2013). Extremophiles and extreme environments. Life Open Access J. 3, 482–485. 10.3390/life3030482 PMC4187170 25369817

[B176] Rasala B. A. Mayfield S. P. (2015). Photosynthetic biomanufacturing in green algae; production of recombinant proteins for industrial, nutritional, and medical uses. Photosynth. Res. 123, 227–239. 10.1007/s11120-014-9994-7 24659086

[B177] Rayko E. Maumus F. Maheswari U. Jabbari K. Bowler C. (2010). Transcription factor families inferred from genome sequences of photosynthetic stramenopiles. New Phytol. 188, 52–66. 10.1111/j.1469-8137.2010.03371.x 20646219

[B178] Reyes-Barrera K. L. Soria-Guerra R. E. López-Martínez R. Huerta L. Salinas-Jazmín N. Cabello-Gutiérrez C. (2021). The entry blocker peptide produced in Chlamydomonas reinhardtii inhibits influenza viral replication *in vitro* . Front. Plant Sci. 12, 641420. 10.3389/fpls.2021.641420 34054890 PMC8149740

[B179] Riaño-Pachón D. M. Corrêa L. G. G. Trejos-Espinosa R. Mueller-Roeber B. (2008). Green transcription factors: a Chlamydomonas overview. Genetics 179, 31–39. 10.1534/genetics.107.086090 18493038 PMC2390610

[B180] Romero-Campero F. J. Perez-Hurtado I. Lucas-Reina E. Romero J. M. Valverde F. (2016). ChlamyNET: a Chlamydomonas gene co-expression network reveals global properties of the transcriptome and the early setup of key co-expression patterns in the green lineage. BMC Genomics 17, 227. 10.1186/s12864-016-2564-y 26968660 PMC4788957

[B181] Saeed M. U. Hussain N. Shahbaz A. Hameed T. Iqbal H. M. N. Bilal M. (2022). Bioprospecting microalgae and cyanobacteria for biopharmaceutical applications. J. Basic Microbiol. 62, 1110–1124. 10.1002/jobm.202100445 34914840

[B182] Salas-Montantes C. J. González-Ortega O. Ochoa-Alfaro A. E. Camarena-Rangel R. Paz-Maldonado L. M. T. Rosales-Mendoza S. (2018). Lipid accumulation during nitrogen and sulfur starvation in Chlamydomonas reinhardtii overexpressing a transcription factor. J. Appl. Phycol. 30, 1721–1733. 10.1007/s10811-018-1393-6

[B183] Sanchez S. Demain A. L. (2017). “Bioactive products from fungi,” in Food bioactives (Cham: Springer), 59–87. 10.1007/978-3-319-51639-4_3

[B184] Sarwar A. Lee E. Y. (2023). Methanol-based biomanufacturing of fuels and chemicals using native and synthetic methylotrophs. Synth. Syst. Biotechnol. 8, 396–415. 10.1016/j.synbio.2023.06.001 37384124 PMC10293595

[B185] Schroda M. (2019). Good news for nuclear transgene expression in Chlamydomonas. Cells 8, 1534. 10.3390/cells8121534 31795196 PMC6952782

[B186] Schroda M. Beck C. F. Vallon O. (2002). Sequence elements within an HSP70 promoter counteract transcriptional transgene silencing in Chlamydomonas. Plant J. 31, 445–455. 10.1046/j.1365-313X.2002.01371.x 12182703

[B187] Schütte G. Eckerstorfer M. Rastelli V. Reichenbecher W. Restrepo-Vassalli S. Ruohonen-Lehto M. (2017). Herbicide resistance and biodiversity: agronomic and environmental aspects of genetically modified herbicide-resistant plants. Environ. Sci. Eur. 29, 5. 10.1186/s12302-016-0100-y 28163993 PMC5250645

[B188] Scranton M. A. Ostrand J. T. Georgianna D. R. Lofgren S. M. Li D. Ellis R. C. (2016). Synthetic promoters capable of driving robust nuclear gene expression in the green alga Chlamydomonas reinhardtii. Algal Res. 15, 135–142. 10.1016/j.algal.2016.02.011

[B189] Seth G. (2012). Freezing mammalian cells for production of biopharmaceuticals. Methods, Curr. Adv. Appl. Cell Cult. 56, 424–431. 10.1016/j.ymeth.2011.12.008 22226818

[B190] Shang C. Pang B. Yu H. Gan S. Li Y. (2022). Identification of targets of transcription factor WRINKLED1-like related to lipid biosynthesis from marine microalga Dunaliella parva. Front. Mar. Sci. 8. 10.3389/fmars.2021.807493

[B191] Shen W. Pan J. Wang G. Li X. (2021). Deep learning-based prediction of TFBSs in plants. Trends Plant Sci. 26, 1301–1302. 10.1016/j.tplants.2021.06.016 34312058

[B192] Shen Z. Bao W. Huang D.-S. (2018). Recurrent neural network for predicting transcription factor binding sites. Sci. Rep. 8, 15270. 10.1038/s41598-018-33321-1 30323198 PMC6189047

[B193] Shi M M. Yu L. Shi J. Liu J. (2022). A conserved MYB transcription factor is involved in regulating lipid metabolic pathways for oil biosynthesis in green algae. New Phytol. 235, 576–594. 10.1111/nph.18119 35342951

[B194] Shin S.-E. Lim J.-M. Koh H. G. Kim E. K. Kang N. K. Jeon S. (2016). CRISPR/Cas9-induced knockout and knock-in mutations in Chlamydomonas reinhardtii. Sci. Rep. 6, 27810. 10.1038/srep27810 27291619 PMC4904240

[B195] Shi Q Q. Chen C. He T. Fan J. (2022). Circadian rhythm promotes the biomass and amylose hyperaccumulation by mixotrophic cultivation of marine microalga Platymonas helgolandica. Biotechnol. Biofuels Bioprod. 15, 75. 10.1186/s13068-022-02174-2 35794631 PMC9261046

[B196] Silveira Júnior A. M. Faustino S. M. M. Cunha A. C. (2019). Bioprospection of biocompounds and dietary supplements of microalgae with immunostimulating activity: a comprehensive review. PeerJ 7, e7685. 10.7717/peerj.7685 31592343 PMC6777487

[B197] Singdevsachan S. K. Auroshree P. Mishra J. Baliyarsingh B. Tayung K. Thatoi H. (2016). Mushroom polysaccharides as potential prebiotics with their antitumor and immunomodulating properties: a review. Bioact. Carbohydr. Diet. Fibre 7, 1–14. 10.1016/j.bcdf.2015.11.001

[B198] Skene P. J. Henikoff S. (2017). An efficient targeted nuclease strategy for high-resolution mapping of DNA binding sites. eLife 6, e21856. 10.7554/eLife.21856 28079019 PMC5310842

[B199] Song J. Zhao H. Zhang L. Li Z. Han J. Zhou C. (2023). The heat shock transcription factor PtHSF1 mediates triacylglycerol and fucoxanthin synthesis by regulating the expression of GPAT3 and DXS in Phaeodactylum tricornutum. Plant Cell Physiol. 64, 622–636. 10.1093/pcp/pcad023 36947404

[B200] Soong Y.-H. V. Coleman S. M. Liu N. Qin J. Lawton C. Alper H. S. (2023). Using oils and fats to replace sugars as feedstocks for biomanufacturing: challenges and opportunities for the yeast Yarrowia lipolytica. Biotechnol. Adv. 65, 108128. 10.1016/j.biotechadv.2023.108128 36921878

[B201] Sproles A. E. Fields F. J. Smalley T. N. Le C. H. Badary A. Mayfield S. P. (2021). Recent advancements in the genetic engineering of microalgae. Algal Res. 53, 102158. 10.1016/j.algal.2020.102158

[B202] Stirk W. A. van Staden J. (2022). Bioprospecting for bioactive compounds in microalgae: antimicrobial compounds. Biotechnol. Adv. 59, 107977. 10.1016/j.biotechadv.2022.107977 35580750

[B203] Sturme M. H. J. Gong Y. Heinrich J. M. Klok A. J. Eggink G. Wang D. (2018). Transcriptome analysis reveals the genetic foundation for the dynamics of starch and lipid production in Ettlia oleoabundans. Algal Res. 33, 142–155. 10.1016/j.algal.2018.05.004

[B204] Sun H. Zhao W. Mao X. Li Y. Wu T. Chen F. (2018). High-value biomass from microalgae production platforms: strategies and progress based on carbon metabolism and energy conversion. Biotechnol. Biofuels 11, 227. 10.1186/s13068-018-1225-6 30151055 PMC6100726

[B205] Sun X.-M. Ren L.-J. Bi Z.-Q. Ji X.-J. Zhao Q.-Y. Huang H. (2018a). Adaptive evolution of microalgae Schizochytrium sp. under high salinity stress to alleviate oxidative damage and improve lipid biosynthesis. Bioresour. Technol. 267, 438–444. 10.1016/j.biortech.2018.07.079 30032058

[B206] Sun X.-M. Ren L.-J. Zhao Q.-Y. Ji X.-J. Huang H. (2018b). Microalgae for the production of lipid and carotenoids: a review with focus on stress regulation and adaptation. Biotechnol. Biofuels 11, 272. 10.1186/s13068-018-1275-9 30305845 PMC6171298

[B253] Südfeld C. Hubáček M. Figueiredo D. Naduthodi M. I. S. van der Oost J. Wijffels R. H. (2021). High-throughput insertional mutagenesis reveals novel targets for enhancing lipid accumulation in Nannochloropsis oceanica. Metab Eng. 66, 239–258. 10.1016/j.ymben.2021.04.012 33971293

[B207] Sydney E. B. Schafranski K. Barretti B. R. V. Sydney A. C. N. Zimmerman J. F. D. Cerri M. L. (2019). Biomolecules from extremophile microalgae: from genetics to bioprocessing of a new candidate for large-scale production. Process Biochem. 87, 37–44. 10.1016/j.procbio.2019.09.012

[B208] Takahashi S. Okubo R. Kanesaki Y. Zhou B. Takaya K. Watanabe S. (2021). Identification of transcription factors and the regulatory genes involved in triacylglycerol accumulation in the unicellular red alga Cyanidioschyzon merolae. Plants 10, 971. 10.3390/plants10050971 34068121 PMC8152781

[B209] Thiriet-Rupert S. Carrier G. Chénais B. Trottier C. Bougaran G. Cadoret J.-P. (2016). Transcription factors in microalgae: genome-wide prediction and comparative analysis. BMC Genomics 17, 282. 10.1186/s12864-016-2610-9 27067009 PMC4827209

[B210] Thiriet-Rupert S. Carrier G. Trottier C. Eveillard D. Schoefs B. Bougaran G. (2018). Identification of transcription factors involved in the phenotype of a domesticated oleaginous microalgae strain of Tisochrysis lutea. Algal Res. 30, 59–72. 10.1016/j.algal.2017.12.011

[B211] Tilman D. (1999). Global environmental impacts of agricultural expansion: the need for sustainable and efficient practices. Proc. Natl. Acad. Sci. 96, 5995–6000. 10.1073/pnas.96.11.5995 10339530 PMC34218

[B212] Torres-Tiji Y. Fields F. J. Mayfield S. P. (2020). Microalgae as a future food source. Biotechnol. Adv. 41, 107536. 10.1016/j.biotechadv.2020.107536 32194145

[B213] Tokunaga S. Sanda S. Uraguchi Y. Nakagawa S. Sawayama S. (2019). Overexpression of the DOF-type transcription factor enhances lipid synthesis in Chlorella vulgaris. Appl. Biochem. Biotechnol. 189, 116–128. 10.1007/s12010-019-02990-7 30877635

[B214] Tolibia S. E. M. Pacheco A. D. Balbuena S. Y. G. Rocha J. López y López V. E. (2023). Engineering of global transcription factors in Bacillus, a genetic tool for increasing product yields: a bioprocess overview. World J. Microbiol. Biotechnol. 39, 12. 10.1007/s11274-022-03460-9 36372802

[B215] Trovão M. Schüler L. M. Machado A. Bombo G. Navalho S. Barros A. (2022). Random mutagenesis as a promising tool for microalgal strain improvement towards industrial production. Mar. Drugs 20, 440. 10.3390/md20070440 35877733 PMC9318807

[B252] Tsai C.-H. Warakanont J. Takeuchi T. Sears B. B. Moellering E. R. Benning C. (2014). The protein Compromised Hydrolysis of Triacylglycerols 7 (CHT7) acts as a repressor of cellular quiescence in Chlamydomonas. Proc. Natl. Acad. Sci. U. S. A. 111 (44), 15833–15838. 10.1073/pnas.1414567111 25313078 PMC4226073

[B216] US Department of energy multi-year program plan (2014). US Department of energy multi-year program plan.

[B217] Van Acker R. C. McLean N. Martin R. C. (2007). “22 - development of quality assurance protocols to prevent GM-contamination of organic crops,” in Handbook of organic food safety and quality, woodhead publishing series in food science, technology and nutrition. Editors Cooper J Niggli U Leifert C (Sawston, United Kingdom: Woodhead Publishing), 466–489. 10.1533/9781845693411.4.466

[B218] Vanholme B. Desmet T. Ronsse F. Rabaey K. Van Breusegem F. De Mey M. (2013). Towards a carbon-negative sustainable bio-based economy. Front. Plant Sci. 4, 174. 10.3389/fpls.2013.00174 23761802 PMC3669761

[B219] Vani M. V. Basha P. O. Chandrasekhar T. Riazunnisa K. (2023). Development and characterization of Chlamydomonas reinhardtii low chlorophyll mutants to improve photosynthetic efficiency and biomass. Braz. J. Bot. 46, 307–318. 10.1007/s40415-023-00887-8

[B220] Varshney P. Mikulic P. Vonshak A. Beardall J. Wangikar P. P. (2015). Extremophilic micro-algae and their potential contribution in biotechnology. Bioresour. Technol., Adv. biofuels Chem. algae 184, 363–372. 10.1016/j.biortech.2014.11.040 25443670

[B221] Vecchi V. Barera S. Bassi R. Dall’Osto L. (2020). Potential and challenges of improving photosynthesis in algae. Plants 9, 67. 10.3390/plants9010067 31947868 PMC7020468

[B222] Veluchamy A. Rastogi A. Lin X. Lombard B. Murik O. Thomas Y. (2015). An integrative analysis of post-translational histone modifications in the marine diatom Phaeodactylum tricornutum. Genome Biol. 16, 102. 10.1186/s13059-015-0671-8 25990474 PMC4504042

[B223] Villegas-Valencia M. González-Portela R. E. de Freitas B. B. Al Jahdali A. Romero-Villegas G. I. Malibari R. (2023). Cultivation of the polyextremophile Cyanidioschyzon merolae 10D during summer conditions on the coast of the Red Sea and its adaptation to hypersaline sea water. Front. Microbiol. 14, 1157151. 10.3389/fmicb.2023.1157151 37152750 PMC10158843

[B224] Wang C. Pfleger B. F. Kim S.-W. (2017). Reassessing *Escherichia coli* as a cell factory for biofuel production. Curr. Opin. Biotechnol. Energy Biotechnol. • Environ. Biotechnol. 45, 92–103. 10.1016/j.copbio.2017.02.010 28292659

[B225] Wang L. Xue C. Wang L. Zhao Q. Wei W. Sun Y. (2016). Strain improvement of Chlorella sp. for phenol biodegradation by adaptive laboratory evolution. Bioresour. Technol. 205, 264–268. 10.1016/j.biortech.2016.01.022 26803904

[B226] Wei L. Xu J. (2018). Optimized methods of chromatin immunoprecipitation for profiling histone modifications in industrial microalgae Nannochloropsis spp. J. Phycol. 54, 358–367. 10.1111/jpy.12623 29444334

[B227] Weirauch M. T. Yang A. Albu M. Cote A. G. Montenegro-Montero A. Drewe P. (2014). Determination and inference of eukaryotic transcription factor sequence specificity. Cell 158, 1431–1443. 10.1016/j.cell.2014.08.009 25215497 PMC4163041

[B228] Wingender E. Dietze P. Karas H. Knüppel R. (1996). TRANSFAC: a database on transcription factors and their DNA binding sites. Nucleic Acids Res. 24, 238–241. 10.1093/nar/24.1.238 8594589 PMC145586

[B229] World Population Prospects (2022). Summary of results. Population Division, Available at: https://www.un.org/development/desa/pd/content/World-Population-Prospects-2022 (Accessed April 12, 23).

[B230] Wösten H. A. B. (2019). Filamentous fungi for the production of enzymes, chemicals and materials. Curr. Opin. Biotechnol., Tissue, Cell Pathw. Eng. 59, 65–70. 10.1016/j.copbio.2019.02.010 30901669

[B231] Wu J. Chen L. Chen M. Zhou W. Dong Q. Jiang H. (2019). The DOF-domain transcription factor ZmDOF36 positively regulates starch synthesis in transgenic maize. Front. Plant Sci. 10, 465. 10.3389/fpls.2019.00465 31031791 PMC6474321

[B232] Wu S. Shi Z. Chen X. Gao J. Wang X. (2022). Arbuscular mycorrhizal fungi increase crop yields by improving biomass under rainfed condition: a meta-analysis. PeerJ 10, e12861. 10.7717/peerj.12861 35178300 PMC8815364

[B233] Xing G. Li J. Li W. Lam S. M. Yuan H. Shui G. (2021). AP2/ERF and R2R3-MYB family transcription factors: potential associations between temperature stress and lipid metabolism in Auxenochlorella protothecoides. Biotechnol. Biofuels 14, 22. 10.1186/s13068-021-01881-6 33451355 PMC7811268

[B234] Xing W. Zhang R. Shao Q. Meng C. Wang X. Wei Z. (2021). Effects of laser mutagenesis on microalgae production and lipid accumulation in two economically important fresh Chlorella strains under heterotrophic conditions. Agronomy 11, 961. 10.3390/agronomy11050961

[B249] Yamaoka Y. Shin S. Choi B. Y. Kim H. Jang S. Kajikawa M. (2019). The bZIP1 Transcription Factor Regulates Lipid Remodeling and Contributes to ER Stress Management in *Chlamydomonas reinhardtii* . Plant Cell 31 (5), 1127–1140. 10.1105/tpc.18.00723 30894460 PMC6533020

[B235] Yang A. Zhang W. Wang J. Yang K. Han Y. Zhang L. (2020). Review on the application of machine learning algorithms in the sequence data mining of DNA. Front. Bioeng. Biotechnol. 8, 1032. 10.3389/fbioe.2020.01032 33015010 PMC7498545

[B236] Yao C. Ai J. Cao X. Xue S. Zhang W. (2012). Enhancing starch production of a marine green microalga Tetraselmis subcordiformis through nutrient limitation. Bioresour. Technol. 118, 438–444. 10.1016/j.biortech.2012.05.030 22717561

[B237] Zaidi S.S.-A. Mahas A. Vanderschuren H. Mahfouz M. M. (2020). Engineering crops of the future: CRISPR approaches to develop climate-resilient and disease-resistant plants. Genome Biol. 21, 289. 10.1186/s13059-020-02204-y 33256828 PMC7702697

[B250] Bai F. Zhang Y. Liu J. (2021). A bZIP transcription factor is involved in regulating lipid and pigment metabolisms in the green alga Chlamydomonas reinhardtii. Algal Research 59, 102450. 10.1016/j.algal.2021.102450

[B238] Zhang B. Wu J. Meng F. (2021). Adaptive laboratory evolution of microalgae: a review of the regulation of growth, stress resistance, metabolic processes, and biodegradation of pollutants. Front. Microbiol. 12, 737248. 10.3389/fmicb.2021.737248 34484172 PMC8416440

[B239] Zhang J. Hao Q. Bai L. Xu J. Yin W. Song L. (2014). Overexpression of the soybean transcription factor GmDof4 significantly enhances the lipid content of Chlorella ellipsoidea. Biotechnol. Biofuels 7, 128. 10.1186/s13068-014-0128-4 25246944 PMC4159510

[B240] Zhang P. Xin Y. He Y. Tang X. Shen C. Wang Q. (2022). Exploring a blue-light-sensing transcription factor to double the peak productivity of oil in Nannochloropsis oceanica. Nat. Commun. 13, 1664. 10.1038/s41467-022-29337-x 35351909 PMC8964759

[B241] Zhang R. Patena W. Armbruster U. Gang S. S. Blum S. R. Jonikas M. C. (2014). High-throughput genotyping of green algal mutants reveals random distribution of mutagenic insertion sites and endonucleolytic cleavage of transforming DNA. Plant Cell 26, 1398–1409. 10.1105/tpc.114.124099 24706510 PMC4036561

[B242] Zhang Y.-H. P. Sun J. Ma Y. (2017). Biomanufacturing: history and perspective. J. Ind. Microbiol. Biotechnol. 44, 773–784. 10.1007/s10295-016-1863-2 27837351

[B243] Zhang Z. Dong J. Ji C. Wu Y. Messing J. (2019). NAC-type transcription factors regulate accumulation of starch and protein in maize seeds. Proc. Natl. Acad. Sci. 116, 11223–11228. 10.1073/pnas.1904995116 31110006 PMC6561305

[B244] Zhao J. Ge Y. Liu K. Yamaoka Y. Zhang D. Chi Z. (2023). Overexpression of a MYB1 transcription factor enhances triacylglycerol and starch accumulation and biomass production in the green microalga Chlamydomonas reinhardtii. J. Agric. Food Chem. 71, 17833–17841. 10.1021/acs.jafc.3c05290 37934701

[B245] Zheng H.-Q. Chiang-Hsieh Y.-F. Chien C.-H. Hsu B.-K. J. Liu T.-L. Chen C.-N. N. (2014). AlgaePath: comprehensive analysis of metabolic pathways using transcript abundance data from next-generation sequencing in green algae. BMC Genomics 15, 196. 10.1186/1471-2164-15-196 24628857 PMC4028061

[B246] Zheng Y. Jiao C. Sun H. Rosli H. G. Pombo M. A. Zhang P. (2016). iTAK: a program for genome-wide prediction and classification of plant transcription factors, transcriptional regulators, and protein kinases. Mol. Plant 9, 1667–1670. 10.1016/j.molp.2016.09.014 27717919

[B247] Zhu Z. Jiang J. Fa Y. (2020). Overcoming the biological contamination in microalgae and cyanobacteria mass cultivations for photosynthetic biofuel production. Molecules 25, 5220. 10.3390/molecules25225220 33182530 PMC7698126

